# Reconstructed Polyamide Nanolayers via Two‐Stage Interfacial Polymerization Engineering for Precise Ion Sieving

**DOI:** 10.1002/advs.74368

**Published:** 2026-02-17

**Authors:** Shuzhen Zhao, Liheng Dai, Feidong Yang, Bowen Li, Pengfei Zhang, Yanyan Liu, Mengyang Hu, Kecheng Guan, Ryosuke Takagi, Hideto Matsuyama

**Affiliations:** ^1^ Research Center for Membrane and Film Technology Kobe University Kobe Japan; ^2^ Department of Chemical Science and Engineering Kobe University Kobe Japan

**Keywords:** interfacial polymerization manipulation, ion sieving, lithium/magnesium separation, polyamide membrane

## Abstract

Efficient lithium extraction from brines remains challenging due to the comparable hydrated radii of Li^+^ and Mg^2+^ and the extreme ionic strength of the feed solutions. In this work, a reconstructed polyamide (PA) nanofiltration membrane was developed via a two‐stage interfacial polymerization strategy, achieving simultaneous structural and electrostatic engineering of the PA selective layer. In the first stage, the relationship between substrate physicochemical properties and the resulting density and stability of the nascent polyamide layer was established through regulation of piperazine (PIP) adsorption‐diffusion behavior. In the second stage, careful selection of a non‐aqueous solvent effectively suppressed acyl chloride hydrolysis and preserved abundant active sites, allowing the successful incorporation of a bidentate quaternary ammonium monomer into the newly formed PA network. This reconstruction generated a confined sub‐nanometer selective layer with a tunable mild positive charge, enabling synergistic steric and electrostatic discrimination between Li^+^ and Mg^2+^. The optimized membrane exhibited excellent Li^+^/Mg^2+^ separation factors that exceeded 60 under diverse operating conditions, while the integrated nanofiltration process achieved nearly 60‐fold lithium enrichment, demonstrating a practical applicability in complex brine matrices. This study establishes a generalizable molecular‐level design reference for co‐ion selective membranes capable of lithium extraction under chemically demanding, high‐ionic‐strength conditions.

## Graphical abstract

A reconstructed polyamide nanofiltration membrane is engineered via a two‐stage interfacial polymerization strategy for lithium extraction from complex brines. Incorporated bidentate quaternary ammonium monomers enable synergistic steric and electrostatic effects within sub‐nanometer channels, achieving 60‐fold lithium enrichment and demonstrating robust practical applicability for high‐precision Li^+^/Mg^2+^ separation.

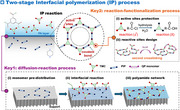

## 1 Introduction

The accelerating development of electric vehicles and renewable energy technologies has driven a substantial increase in global lithium (Li) demand, as Li serves as a pivotal component in advanced batteries and sustainable energy storage systems [1, 2]. Salt‐lake brines, which account for approximately 66% of global Li reserves, are considered strategic resources [3, 4], while their inherently high magnesium‐to‐lithium (Mg^2+^/Li^+^) mass ratios, usually exceeding 50, propose severe challenges for conventional extraction methods such as solar evaporation and chemical precipitation [5, 6]. Meanwhile, similar ionic radii (only ∼0.5 Å difference) between Li^+^ and Mg^2+^ also require the extraction process to possess precise ion sieving ability [7, 8]. To address these challenges, compared to traditional extraction technologies, nanofiltration (NF) membranes enable continuous, energy‐efficient processing with reduced chemical usage and improved scalability, making them highly attractive for lithium extraction from complex brines [9, 10].

NF membranes represented by polyamide (PA), typically formed by interfacial polymerization (IP) between piperazine (PIP) and trimesoyl chloride (TMC), are widely used in water desalination and resource recovery [11, 12]. Notably, current PA membranes fabricated by PIP and TMC usually show negative charge, difficult to distinguish the ions only according to the Donnan effect [13], while designing a strong positive charged membrane can repulsive high valence ions but also significantly reject Li^+^, resulting in low Li^+^ recovery [14, 15, 16]. Meanwhile, although narrowing the pore size distribution of PA membrane can achieve superior Li^+^ selectivity, the adjusting process usually requires fine operation or chemical dosage, and it may tend to increase the water transport resistance [17]. Therefore, it is full of challenges to achieve the balance between surface charge and channel size to simultaneously ensure high Li^+^ flux and selective ion separation [18].

**FIGURE 1:**
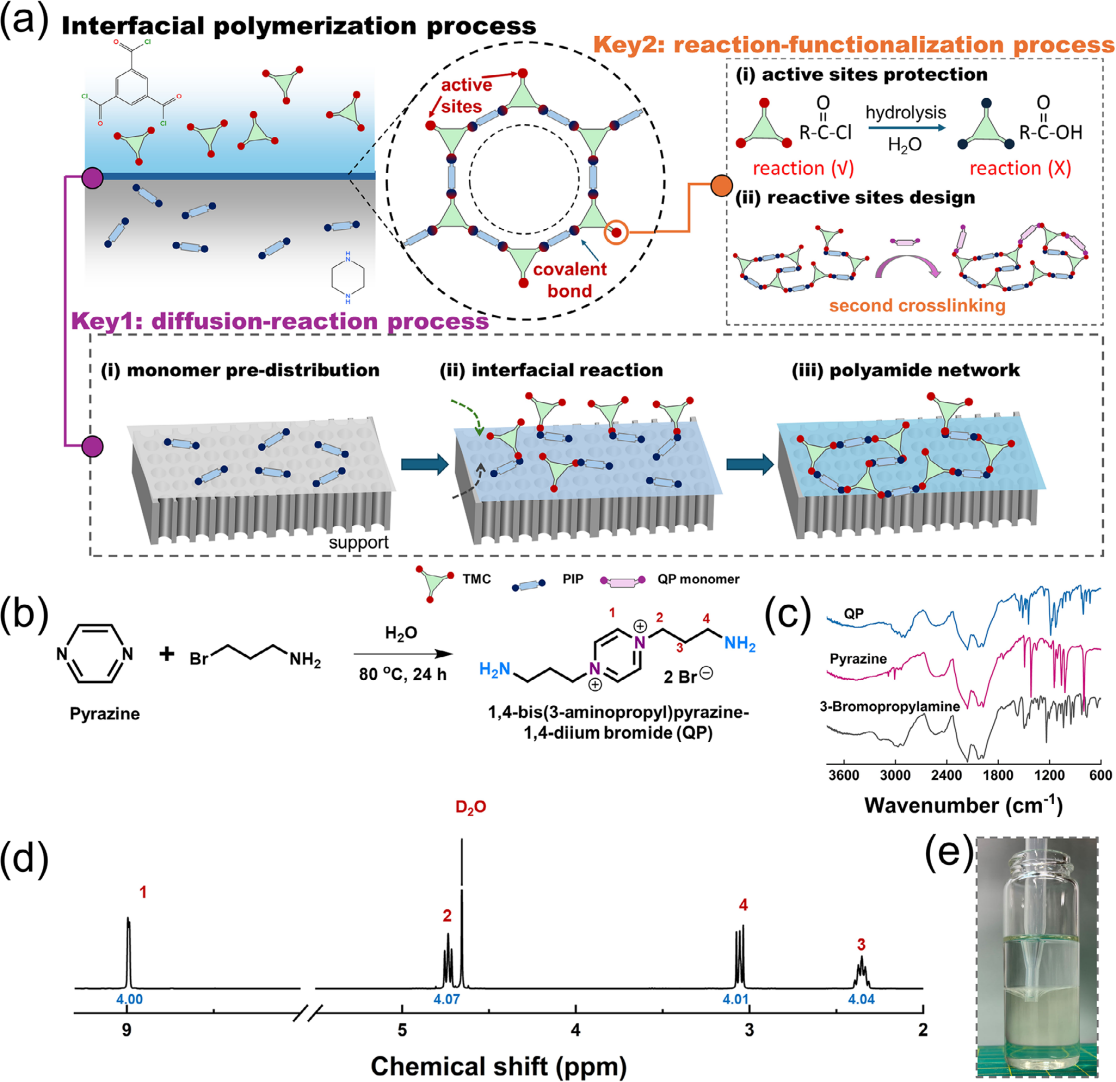
Two‐stage IP process and QP monomer characterization (a) keys for the preparation process of the PA membranes via the two‐stage IP reaction, (b) Illustration of QP monomer synthesis reaction, (c) FTIR spectra and (d) ^1^H NMR spectra of QP monomer, (e) Digital image of free‐standing film via interfacial polymerization between QP aqueous solution (1.0 wt.%, bottom) and TMC hexane solution (0.1 wt.%, top).

The two‐stage IP process is a promising method to manipulate the PA network and surface chemistry, including initial PA nanolayer construction and second‐stage PA nanolayer modification/functionalization [19, 20, 21]. To further make the membrane satisfy the precise separation requirement, two key issues should be addressed: (i) stable PA layer construction (two‐stage IP process usually loose the whole membrane structure to further widen the pore size distribution); (ii) sufficient reaction active sites maintenance (hydrolysis of TMC will end up the further IP reaction) [22, 23], as shown in Figure 1a. Therefore, it is essential to construct a stable initial PA nanolayer and preserve abundant active sites. In addition, a charge monomer with reactive groups should be rationally designed. These strategies enable simultaneous regulation of charge distribution and pore‐size confinement, thereby balancing ion selectivity and water permeability for lithium extraction from brines.

In this study, a reconstructed PA membrane with structurally and electrostatically engineering was developed by a designed bidentate quaternary ammonium monomer, 1,4‐bis(3‐aminopropyl) pyrazine‐1,4‐diium bromide (labeled QP) based on the two‐stage IP process (Figure 1b). The key parameters of support properties to construct a stable PA nanofilm were systematically elucidated and active sites of initial PA nanolayer were finely controlled for further reconstruction of the separation layer. This design optimizes the surface chemistry while preserving the channel sieving ability of the PA network. The resulting optimum membrane demonstrated excellent separation performance achieving Mg^2+^ rejection above 97% and a Li^+^/Mg^2+^ separation factor exceeding 60. And the water flux reached approximately 2.5 times that of the pristine PA membrane. Hence, the integration of the two‐stage IP strategy with tailored charge‐regulating monomers provides a feasible pathway to simultaneously stabilize the polyamide nanostructure and modulate the surface electrostatics. This dual regulation enables precise pore size confinement and charge distribution control, thereby establishing a robust structural and functional reference for next‐generation nanofiltration membranes with superior Li^+^/Mg^2+^ selectivity and high‐water permeability.

## 2 Results and Discussion

A reactive functional monomer is the key for the two‐stage IP process and PA structural engineering manipulation. Firstly, as shown in Figure 1b, a quaternary ammonium monomer QP is designed and successfully synthesized, which has two amine groups as reaction sites and possesses a quaternary ammonium altered benzene ring. H nuclear magnetic resonance (^1^H NMR) and Fourier transform infrared (FTIR) spectroscopy confirm the successful construction of this special chemical structure (Figure 1c,d). Importantly, it can directly form a uniform and continuous film within 1 min upon reaction with TMC, similar to the reaction between PIP and TMC (Figure 1e; Figure S1a), as evidenced by the distinct amide absorption band at 1640 cm^−1^ in the ATR‐FTIR spectrum (Figure S1b).

Different from the common construction units, PIP, the prepared nanofilm (TMC‐QP) shows excessively high LiCl retention and low flux (Figure S2), which cannot satisfy the requirement of high Li recovery for NF membrane. This behavior is largely attributed to the strong positive charge of the quaternary ammonium groups, which induces repulsive interactions, as well as the steric hindrance and pore densification resulting from their larger molecular structure [24, 25]. Consequently, the TMC‐QP membrane is still limited by the inherent trade‐off between water flux and ion selectivity. Therefore, we redefine a two‐stage IP strategy, which aims to combine the intrinsic selectivity of the conventional PA layer with the interfacial tuning advantages of the quaternary ammonium functional layer, thereby overcoming the traditional trade‐off.

**FIGURE 2:**
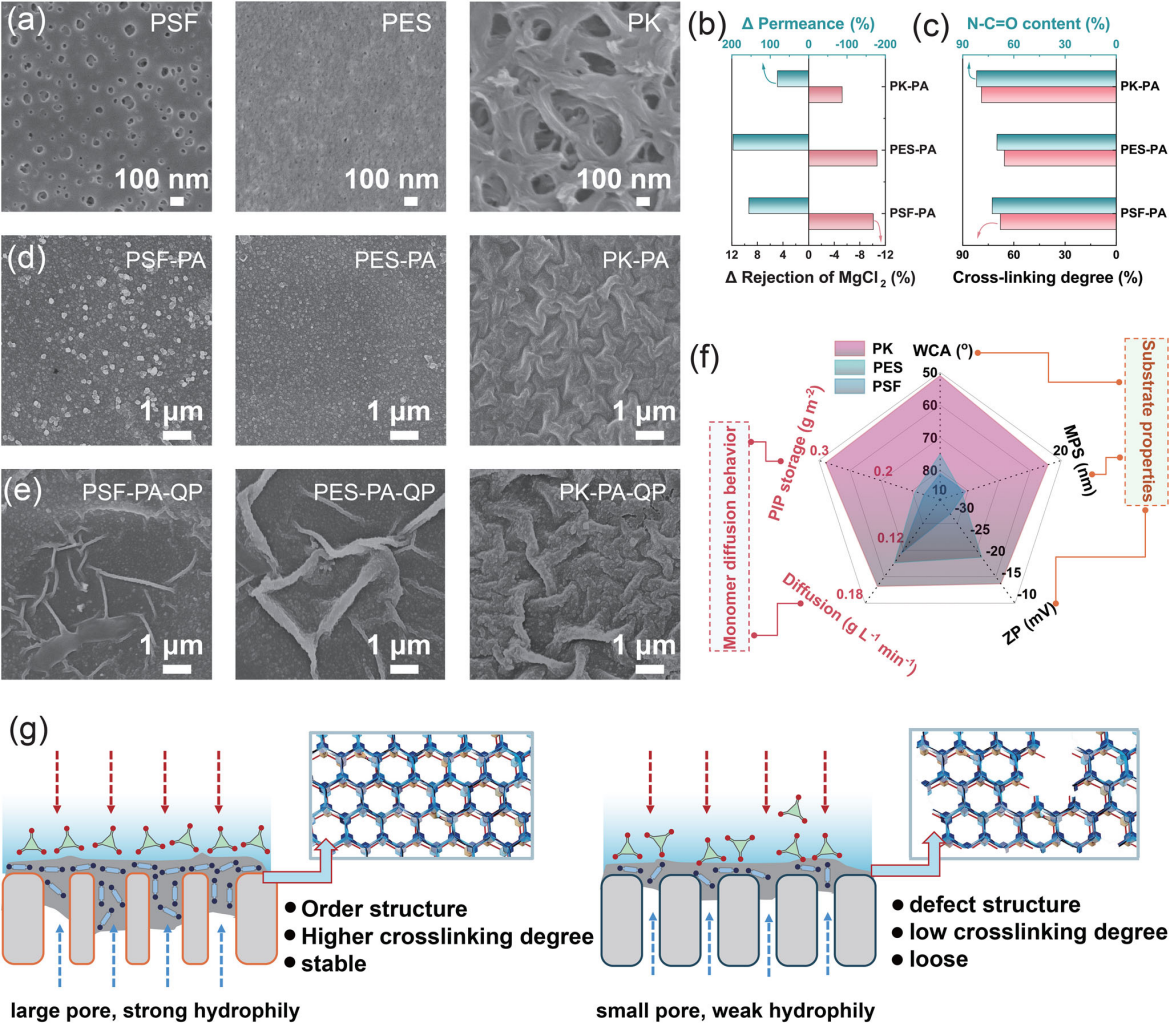
Membrane performance and surface characteristics for different supports. (a) SEM images of the support surfaces (PSF, PES, PK); (b) Changes in MgCl_2_ rejection and water permeance after QP two‐stage IP on different supports; (c) Crosslinking degree and amide bond fraction of PA layers formed by primary IP on different supports; SEM images of (d) the PA layers formed by primary IP (PSF‐PA, PES‐PA, PK‐PA) and (e) the QP‐modified PA membranes on different supports (PSF‐PA‐QP, PES‐PA‐QP, PK‐PA‐QP). (f) Radar chart of physicochemical properties of different supports (WCA means water contact angle, MPS means mean pore size, and ZP means surface zeta potential); (g) Schematic diagram of the influence of different supports on selective layer formation.

In the two‐stage IP process, the PA layer formed in the first step serves as the foundational separation layer, and its stability is crucial for achieving precise ion separation. However, the functional monomers or introduced solvents in the second functionalization step may induce interface disturbance or swelling, leading to structural loosening, which weakens its inherent rejection capability and ultimately degrades the final separation performance. Therefore, it is important to construct a stable initial PA layer, which is closely related to the reaction‐diffusion kinetics of PIP and TMC dominated by support properties [12]. To reveal and achieve the successful construction of an initial stable PA layer, three support membranes with different properties were employed, including two common commercial substrates, polyethersulfone (PES), polysulfone (PSF), and laboratory‐cast polyketone (PK) membrane (Figure 2a).

The separation performance of the as‐prepared PA membranes formed on different supports was then evaluated. As shown in Figure S3, all PA membranes exhibited high MgCl_2_ rejection (>90%), while notably the PK‐based membrane achieved the best performance with 97.9% MgCl_2_ rejection and nearly 40% LiCl rejection, which might be attributed to its denser and more uniform selective layer. However, all membranes showed relatively low pure‐water permeance (≤6.5 L·m^−2^·h^−1^·bar^−1^), reflecting the typical trade‐off between high rejection and low flux in conventional NF membranes. As mentioned above, a two‐stage IP process was performed using an aqueous solution of the bidentate quaternary ammonium monomer QP to address the trade‐off limitation (Figure S4). As shown in Figure 2b and Figure S5, two‐stage IP process based on QP monomer markedly enhanced the water permeance towards all membranes, whereas their MgCl_2_ rejection decreased to some extent. It can be seen that the first‐formed PA layer structures were influenced during second IP process. Notably, the PK‐based membrane exhibited the smallest loss in MgCl_2_ rejection while achieving a substantial flux enhancement due to the stability of the pristine PA layer, thereby offering an excellent balance between permeability and selectivity. To further explain the origin of the superior performance of the PK‐based membrane after QP modification, X‐ray photoelectron spectroscopy (XPS) was employed to quantify the properties of the primary PA layers formed during the initial IP. As shown in Figure 2c, Figure S6 and Table S1, the PA layer on the PK support exhibited the highest cross‐linking degree and amide bond fraction, indicating the densest structure among the samples. Furthermore, the PA layers on PES and PSF supports exhibited typical nodular structures, whereas the PA layer on the PK support displayed a uniform and fine wrinkled morphology (Figure 2d). After two‐stage IP, these morphological differences became more pronounced. The PA layers on PES and PSF supports began to exhibit localized wrinkling, while the PA layer on the PK support showed a marked increase in both the amplitude and scale of the wrinkles (Figure 2e).

Following the significant changes in membrane performance and morphology, it can be clearly seen that the support membranes play an important role in the construction of initial stable PA layer. To comprehensively evaluate their effect, we systematically characterized key physicochemical parameters of these supports, including surface charge, hydrophilicity, roughness, pore size, and pure water flux. As shown in Figure 2f and Figures S7–S9, the PK support exhibited a larger pore size and superior hydrophilicity compared to its commercial support where these properties determine the good distribution of monomers at the interface and the reaction kinetics to ultimately govern the structure of the PA layer [26, 27, 28]. To reveal this, the adsorption and diffusion behavior of PIP on different supports was monitored and analyzed [29]. As shown in Figure 2f and Figure S10, compared with the PES and PSF supports, the PK membrane exhibited approximately twice the PIP adsorption capacity, attributable to its superior hydrophilicity and larger pore size, while the diffusion rate showed no significant difference among the three supports. The enhanced adsorption ensures a higher local concentration of aqueous monomers at the reaction interface, promoting more extensive cross‐linking with the organic phase monomers and thus forming a denser polyamide network. Additionally, the uniform distribution of adsorbed monomers across the hydrophilic surface minimizes defects and irregularities, resulting in a more consistent and selective layer structure (Figure 2g). Therefore, the substrate not only provides mechanical support but also fundamentally regulates the IP process through its influence on monomer adsorption, diffusion, and subsequent reaction.

**FIGURE 3:**
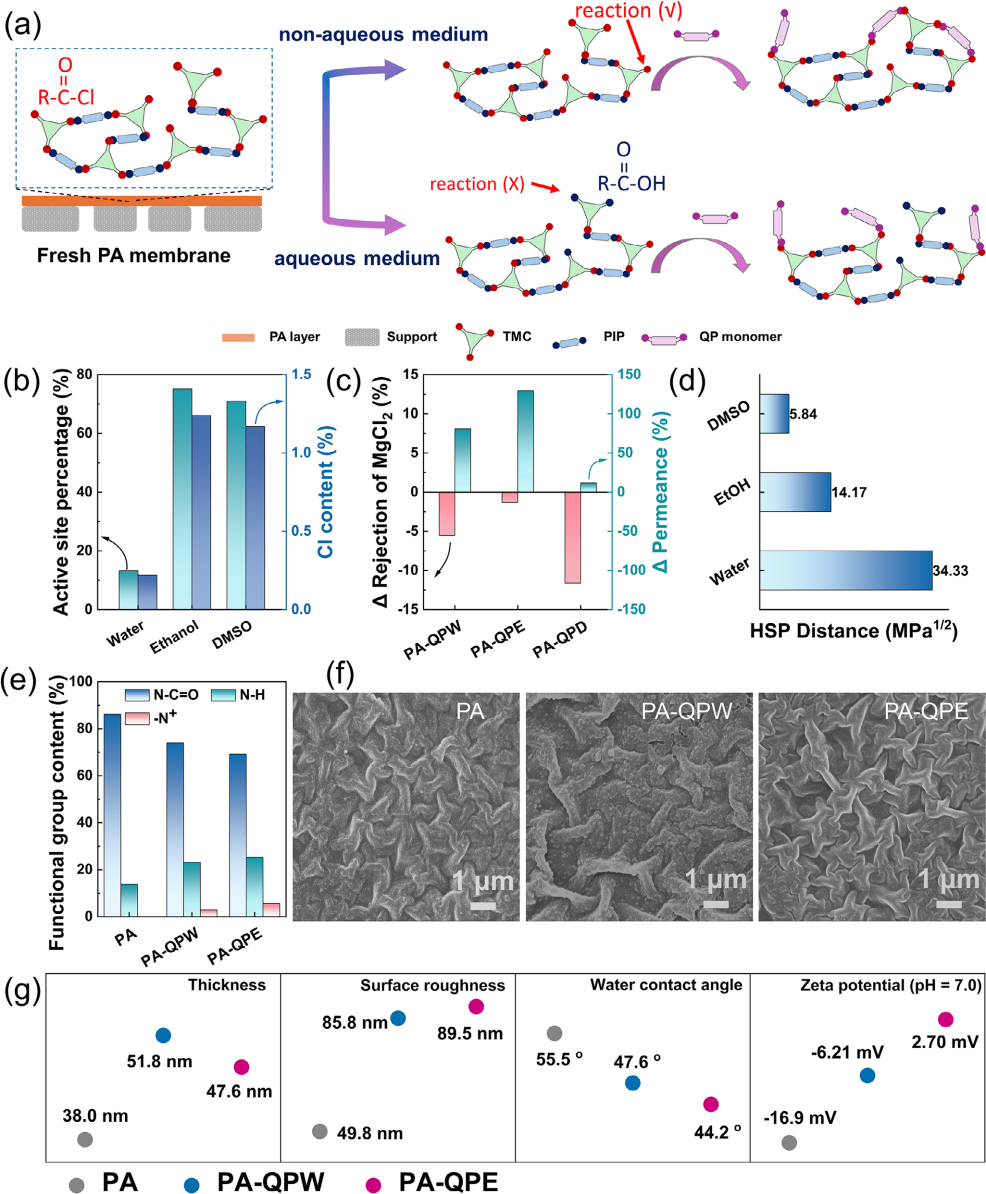
Non‐aqueous two‐stage interfacial polymerization strategy. (a) Schematic diagram of non‐aqueous two‐stage interfacial polymerization; (b) Active reaction site percentages for two‐stage interfacial polymerization under different solvents; (c) Change rates of MgCl_2_ rejection and water permeance for PA‐QP membranes prepared using different solvent systems; (d) differences in Hansen solubility parameter (Ra) between solvents and the PA layer; (e) Surface functional group content; (f) Surface SEM images; (g) Active layer thickness, surface roughness, water contact angle and zeta potential (pH 7.0) of PA, PA‐QPW, and PA‐QPE membranes. Note: the membranes optimized via two‐stage interfacial polymerization in ethanol, DMSO, and aqueous systems are hereafter referred to as PA‐QPE, PA‐QPD, and PA‐QPW, respectively.

Based on a stable PA layer, the success of the following second‐stage IP relies critically on the effective control of reactive sites present on the primary PA surface, mainly unreacted acyl chloride groups. Premature hydrolysis of these groups significantly compromises their reactivity, thereby limiting the extent of polymerization and hindering further improvements in membrane performance. In the aqueous system used previously, residual acyl chloride groups on the membrane surface inevitably underwent hydrolysis, substantially reducing the number of active sites available for subsequent reactions [18, 19]. To address this issue, the QP concentration in the aqueous phase was first optimized to evaluate whether tuning monomer availability could improve performance (Figure S11). However, the overall performance remained constrained by the inherent limitations of the unavoidable hydrolysis of acyl chloride groups in aqueous environments, reducing the ability of precise ion sieving. Alternatively, changing the solvent condition from aqueous to organic might suppress the hydrolysis of acyl chloride groups, thereby preserving more reactive sites for further reactions (Figure 3a). Ethanol and dimethyl sulfoxide (DMSO) were selected as representative non‐aqueous solvents, as they can dissolve the monomers without triggering hydrolysis, providing a stable interface for the second‐stage reaction.

To prove the preservation of reactive sites, energy dispersive X‐ray spectroscopy (EDS) analysis was conducted and analyzed. By defining the relative retention of reactive sites as the ratio of the Cl content measured after different treatments to this initial baseline, using ethanol and DMSO as solvents, the percentage of active sites available for two‐stage IP was significantly higher than in water. The results demonstrated that non‐aqueous solvents can markedly reduce the hydrolysis of acyl chlorides, thereby providing more reactive sites for subsequent reconstruction of PA layers (Figure 3b; Figure S12). Therefore, two‐stage IP reaction with 2 wt.% QP was conducted in ethanol and DMSO systems, and the resulting membranes were evaluated. As shown in Figure 3c and Figure S13, the ethanol‐based membrane showed both high selectivity and a 2.5‐fold increase in water permeance, while the membrane treated with DMSO exhibited unexpectedly poor performance with low selectivity and permeance. This result deviated from the expected trend for non‐aqueous solvents. To further illustrate the solvent effect on the PA layer, the Hansen solubility parameter (HSP) framework correlating the similarity of solubility parameters with the solubility of polymers in solvents was calculated and compared. Using the HSPiP software (***6.1.02***), we calculated the HSP values for PA chain segments and determined the HSP Distance (Ra) values between the PA layer and different solvents (Figure 3d; Figures S14, S15 and Table S2). The results showed that DMSO had the smallest Ra value relative to the PA layer, indicating the strongest affinity and the greatest propensity to induce swelling or partial dissolution of the PA layer [30, 31]. Its strong polarity and high hydrogen‐bond acceptor capacity allow DMSO molecules to penetrate the nascent polyamide layer and strongly interact with polymer chains, disrupting chain packing, inducing swelling and structural rearrangement, and ultimately compromising the density and integrity of the selective barrier. Furthermore, DMSO at the interface suppressed rapid film curing, leading to a looser second‐stage layer with structural defects [31, 32]. This finding clearly demonstrates that not all non‐aqueous solvents are suitable for reactive site modulation; solvent selection must comprehensively consider its interactions with the PA layer, particularly the potential for swelling or dissolution. Therefore, we selected ethanol as non‐aqueous solvent for the following reconstruction of PA layer and the concentration of QP in ethanol for the two‐stage IP was also investigated (Figure S16). For clarity in the following discussion, the membranes optimized via two‐stage interfacial polymerization in ethanol and aqueous systems are hereafter referred to as PA‐QPE and PA‐QPW, respectively.

To further elucidate the effect of QP incorporation under different solvent conditions on the surface structure and properties of the membranes, systematic characterizations were conducted. First, as shown in Figure S17, all membranes (PA, PA‐QPW, and PA‐QPE) exhibited a characteristic amide bond absorption at 1640 cm^−1^, indicating the good preservation of the main PA skeleton. Further XPS analysis confirmed that the successful reaction of QP with residual acyl chlorode groups and functionalization on membrane surface due to the appearance of Br signal and the notable changes in O and N elemental contents (Figure S18) [33]. Meanwhile, a new quaternary ammonium peak (N^+^) at 402.5 eV and changes in the proportions of N─H and C═O─N peaks were observed to further confirm the successful surface chemical properties reconstruction of PA layers via the two‐stage IP (Figure 3e; Figure S19). Notably, the PA‐QPE membrane exhibited a higher Br content than PA‐QPW, suggesting that a greater amount of QP monomer participated in the reaction in the non‐aqueous solvent system. Following SEM and AFM analysis indicated that the membrane surface morphologies were also reconstructed after QP two‐stage modification, showing significantly larger surface wrinkle dimensions and increased surface roughness compared with the pristine PA membrane (Figure 3f; Figure S20). Notably, after reacting with QP, the thickness of PA membrane increased for PA‐QPW and PA‐QPE due to the diffusion and reaction of QP monomers at the surface and interface of the original PA layer. The thickness differences between PA‐QPW and PA‐QPE further confirm the solvent effect on the efficiency of the second‐stage IP based on the amount of residual acyl chloride groups (Figure 3g; Figure S21). In addition, the intrinsic hydrophilicity of QP also optimizes the surface wetting properties of reconstructed membranes [34, 35], showing decreased water contact angle compared to the pristine PA membrane (Figure 3g; Figure S22).

Importantly, the surface negative charge of the membranes was substantially reduced after QP modification, with PA‐QPW approaching neutrality and PA‐QPE exhibiting a slight positive charge (Figure 3g; Figure S23). This surface charge regulation enhanced electrostatic repulsion against divalent cations while the moderate positive charge and more uniform nanostructure of PA‐QPE minimized monovalent cation retention, collectively enabling precise discrimination between mono‐ and divalent cations. Notably, membranes reconstructed with QP under different solvent environments exhibited distinct ion separation tendencies, as shown in Figures S11, S16, and S24. While both types of membranes demonstrated appreciable selectivity toward mono‐ and divalent ions across both anionic and cationic species, preferential behavior was evident: PA‐QPW membranes showed enhanced efficiency in anion separation, whereas PA‐QPE membranes displayed superior cation selectivity. Since their pore size and distribution were largely comparable, the divergence can be primarily ascribed to differences in surface charge, a conclusion consistent with the observed separation performance. These results underscore that the solvent environment governs the preservation of reactive sites and the structural reconstruction of the PA network to further modulate surface charge properties, thereby deciding the directional preference of ion selectivity.

The PA‐QPE membrane exhibited only a slight change in MgCl_2_ rejection compared to the pristine PA membrane, while its water permeance increased significantly from 6 to approximately 15 L·m^−2^·h^−1^·bar^−1^, reaching about twice that of the unmodified membrane. This improvement arises from a synergistic mechanism involving channel structural homogenization and electrostatic regulation. On the one hand, the solvent used during QP grafting induces partial swelling of the newly formed PA layer, generating a temporarily loosened porous structure that facilitates water transport. Subsequently, residual acyl chloride groups react with the primary amines of QP molecules, forming covalent bonds and locally densifying the network via molecular bridging. Furthermore, the introduction of quaternary ammonium groups significantly increases the positive surface charge density, which enhances electrostatic repulsion against multivalent cations such as Mg^2+^. This synergistic interplay of swelling‐induced structural reconstruction, localized bridging, and charge modulation enables the PA‐QPE membrane to achieve a substantial increase in water flux while maintaining robust divalent ion rejection.

**FIGURE 4:**
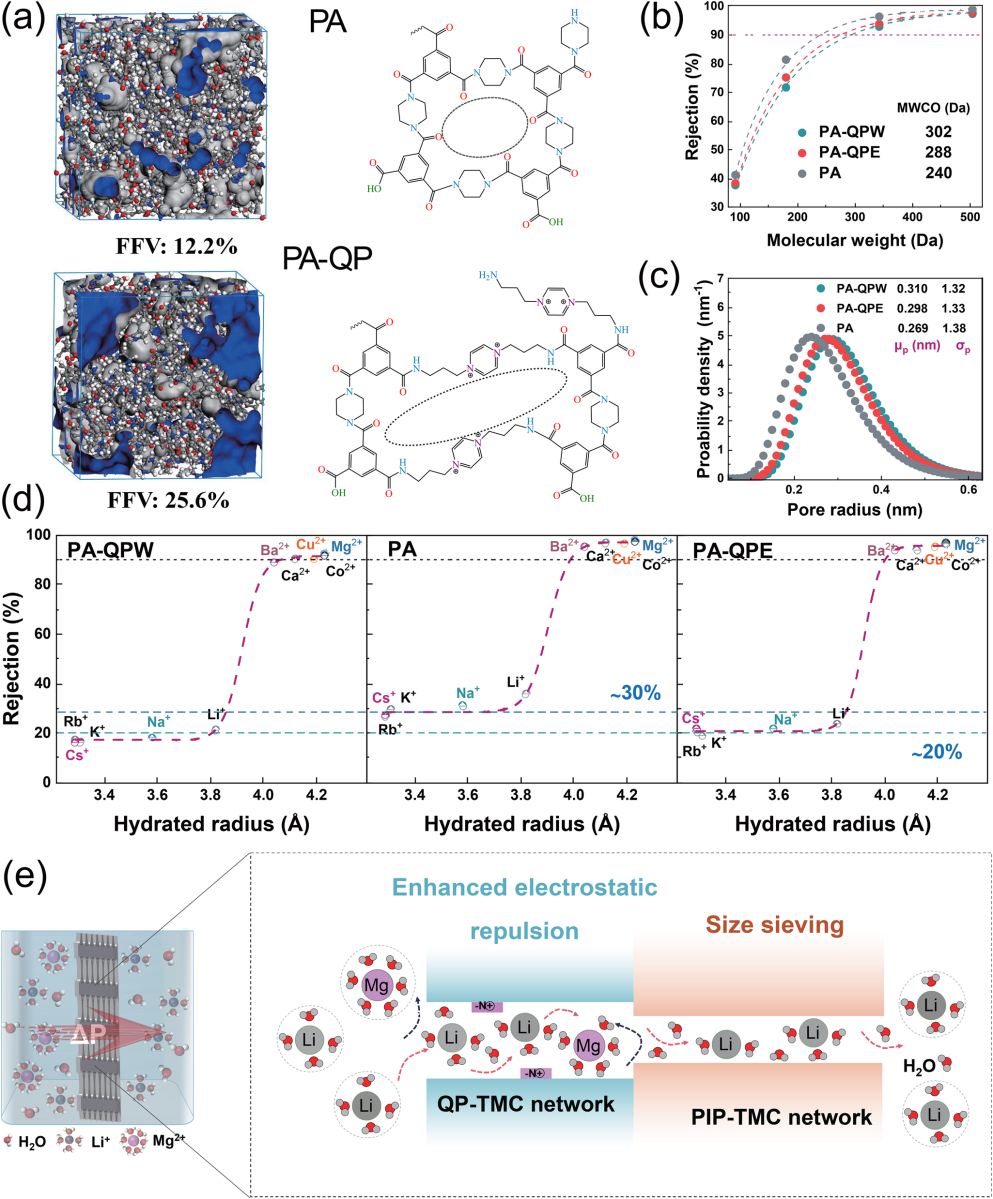
Effects of QP two‐stage interfacial polymerization on membrane structure and ion separation. (a) Probable crosslinked structure of the PA and PA‐QP nanofilms and 3D visualization of the microstructure in the amorphous regions of PA and PA‐QP polymer networks; (b) Molecular weight cut‐off (MWCO) curves; (c) Pore size distribution of different memnranes; (d) Retention curves for cations with different hydrated radii; (e) Schematic diagram of nanofiltration membranes for precise separation of magnesium and lithium.

Molecular dynamics (MD) simulations were performed using simulated polymerization algorithms to construct crosslinked polymer network models to investigate channel structure change (Figure 4a). The fractional free volume (FFV) was then computed from equilibrated structures to quantify internal porosity and interchain packing characteristics. The pristine PA membrane exhibited a relatively compact structure with an FFV of approximately 11.3%, whereas the QP‐modified PA‐QP membrane showed a significantly higher FFV of 23.6%. This notable increase indicates that the introduction of QP during two‐stage interfacial polymerization will induce PA chains rearrangement to produce a more open network architecture with greater internal free volume. The expanded FFV facilitates the formation of additional permeation pathways, thereby contributing to enhanced water flux. Furthermore, the results of molecular weight cutoff (MWCO) showed membranes modified through two‐stage IP exhibited larger MWCO values, reaching 302 Da for PA‐QPW and 288 Da for PA‐QPE, both notably higher than the 240 Da measured for the unmodified PA membrane (Figure 4b). Meanwhile, the average pore size of PA‐QPW and PA‐QPE was also increased compared to initial PA membrane, but their pore size distribution became more uniform (Figure 4c). These results indicate that the two‐stage IP process involving QP not only enlarged the effective pore size but also enhanced structural uniformity, which facilitates monovalent cation transport while maintaining separation precision.

To verify the synergistic contribution of structural and electrostatic regulation, single‐salt rejection experiments were conducted for cations with varying hydrated radii, as shown in Figure 4d and Table S3. The unmodified PA membrane exhibited high rejection rates exceeding 94% for divalent cations, while allowing approximately 30% retention of monovalent cations. Following two‐stage interfacial polymerization with quaternary ammonium monomers, the reconstructed membranes, particularly PA‐QPE, exhibited significantly reduced retention of monovalent cations to below 20%, while maintaining high rejection of divalent cations, indicating excellent selectivity between monovalent and divalent species. Employing the Stokes radius as the descriptor also shows an identical separation trend (Figure S25), further validating that the observed selectivity originates from the coupled steric‐hindrance and electrostatic‐repulsion mechanism. QP functionalization during second stage IP slightly enlarges the average pore radius while narrowing its distribution, which facilitates the water permeation but simultaneously maintains a high‐resolution ion sieving ability. Meanwhile, the quaternary ammonium groups endow the membrane surface with a mild positive charge that enhances Donnan exclusion of divalent cations (Figure 4e).

**FIGURE 5:**
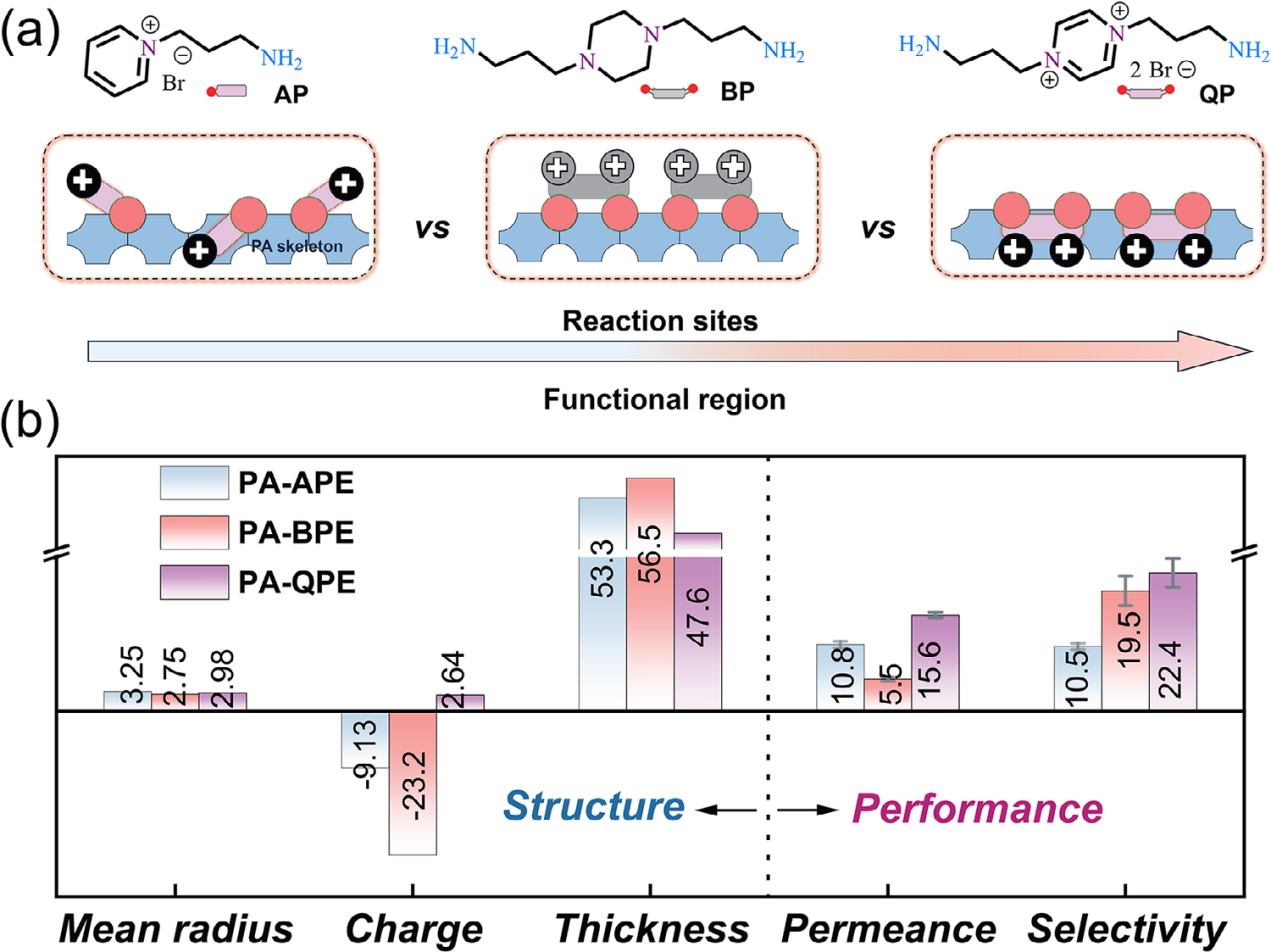
Verification of the bidentate QP strategy. (a) Chemical structures of QP and control monomers (AP and BP) and their reaction pathways during the two‐stage IP. (b) Summary comparison of key structural parameters (mean pore radius (Å), surface charge (mV) when pH was 7.0, and active layer thickness (nm)) and separation performance (pure water permeance (LMH/bar) and single salt selectivity (‐)) of PA‐APE, PA‐BPE, and PA‐QPE membranes.

Furthermore, the reconstruction process of PA membrane induced by active monomer during second stage IP was also required to be finely designed to satisfy the high ion sieving ability. Therefore, another two monomers were selected and synthesized, including the monodentate quaternary ammonium (AP) and the diamine monomer (BP), for comparison with the bidentate quaternary ammonium QP (Figure 5a; Figure S26). Similarly, both selected monomers can well react with residual acyl chloride groups to reconstruct the initial PA layer based on the ethanol system (Figure S27). However, the difference in reaction sites and functional regions of the three monomers induced different PA layer reconstruction results. AP with mono site can only induce limited regrowth but it failed to achieve the recrosslinking, resulting in looser and thicker membrane structure. Compared to QP with rigid structure and positively charged sites, BP with dual sites promotes outward second growth with residual acyl chlorides, resulting in the thickest PA layers (Figure S28). Meanwhile, its dual sites can still induce secondary crosslinking and the flexible hexatomic ring facilitated the formation of more twisty polymer chains (Figure 5a). As shown in Figure 5b and Figure S29, PA‐BPE possessed the smallest average channel size, and the corresponding permeance was also the lowest. In addition, functional region for monomers endowed the reconstructed membrane different surface charge. Due to the existence of the quaternary ammonium group, the introduction of AP and QP weakened the negative charge of initial PA layer, while BP without strong positive dominant made the reconstructed membrane still maintain negative charge. In comparison, QP, influenced by its quaternary ammonium center and rigid structure, primarily undergoes local rearrangement rather than vertical thickening within the nascent PA network, giving rise to the smallest thickness change [33, 36]. The resultant PA‐QPE membrane showed the optimum water permeance and selectivity of LiCl/MgCl_2_. Briefly, these results demonstrate that QP enables synergistic control of PA network structure and charge distribution during the two‐stage IP, enabling PA‐QPE to achieve the most favorable balance between water permeability and Li^+^/Mg^2+^ selectivity. In contrast, AP provides limited structural tuning, and BP promotes excessive densification that compromises selective ion transport, highlighting the critical role of bidentate quaternary ammonium design in achieving high‐performance PA membranes.

After successfully reconstructing the initial PA layer by QP monomer based on two‐stage IP process, further binary salt filtration experiments using LiCl and MgCl_2_ mixtures were conducted to assess the separation behavior of the PA‐QPE membrane. As shown in Figure S30a, the PA‐QPE membrane maintained outstanding Li^+^/Mg^2+^ selectivity across a broad range of operating conditions, with separation factors consistently exceeding 60, while achieving approximately 2.5‐fold higher water permeance than the unmodified PA membrane. This simultaneous enhancement in flux and selectivity reflects the synergistic effects of the optimized structural and interfacial properties introduced by two‐stage interfacial polymerization. In contrast, the PA‐QPW membrane prepared in aqueous media exhibited substantially reduced selectivity and permeance under identical conditions (Figure S30b), underscoring the superior role of non‐aqueous solvents in enabling compact network formation and favorable charge regulation during QP incorporation.

Importantly, when the Mg^2+^/Li^+^ feed mass ratio varied from 20 to 60 and the total ion concentration ranged from 2000 to 6000 ppm, the PA‐QPE membrane retained a stable Li^+^/Mg^2+^ separation factor between 60 and 70, demonstrating robust structural and electrostatic stability even under chemically dynamic feed conditions. Mechanistically, the high Mg^2^^+^ rejection of PA‐QPE results from the cooperative interplay of sub‐nanometer pore and fine‐tuned surface charge effects. Mg^2+^, with a larger hydrated radius (0.43 nm) and significantly higher hydration energy (1900 kJ/mol) compared to Li^+^ (0.38 nm, 500 kJ/mol), experiences substantial energy barriers to dehydration and transmembrane transport [37, 38]. Meanwhile, the incorporation of quaternary ammonium‐functionalized QP molecules produces a compact, positively charged selective layer that enhances steric hindrance for larger ions and strengthens electrostatic repulsion against divalent cations, thereby generating a highly discriminative size‐charge hybrid sieving mechanism. In contrast, Li^+^ exhibits a notably different behavior. Under certain test conditions, a negative rejection of Li^+^ was observed, where its concentration in the permeate exceeded that in the feed [39, 40]. The observed negative rejection of Li^+^ is fundamentally attributed to the counter‐ion competition mechanism [41], in which strongly hydrated Mg^2+^ accumulates near the membrane pores and alters the local hydration environment. This accumulation promotes the partial dehydration of the weakly hydrated monovalent Li^+^ at the pore entrance, effectively reducing their hydration radius and the associated steric exclusion. Consequently, Li^+^ experiences lower transport resistance and exhibits enhanced permeability across the membrane [42, 43].

**FIGURE 6:**
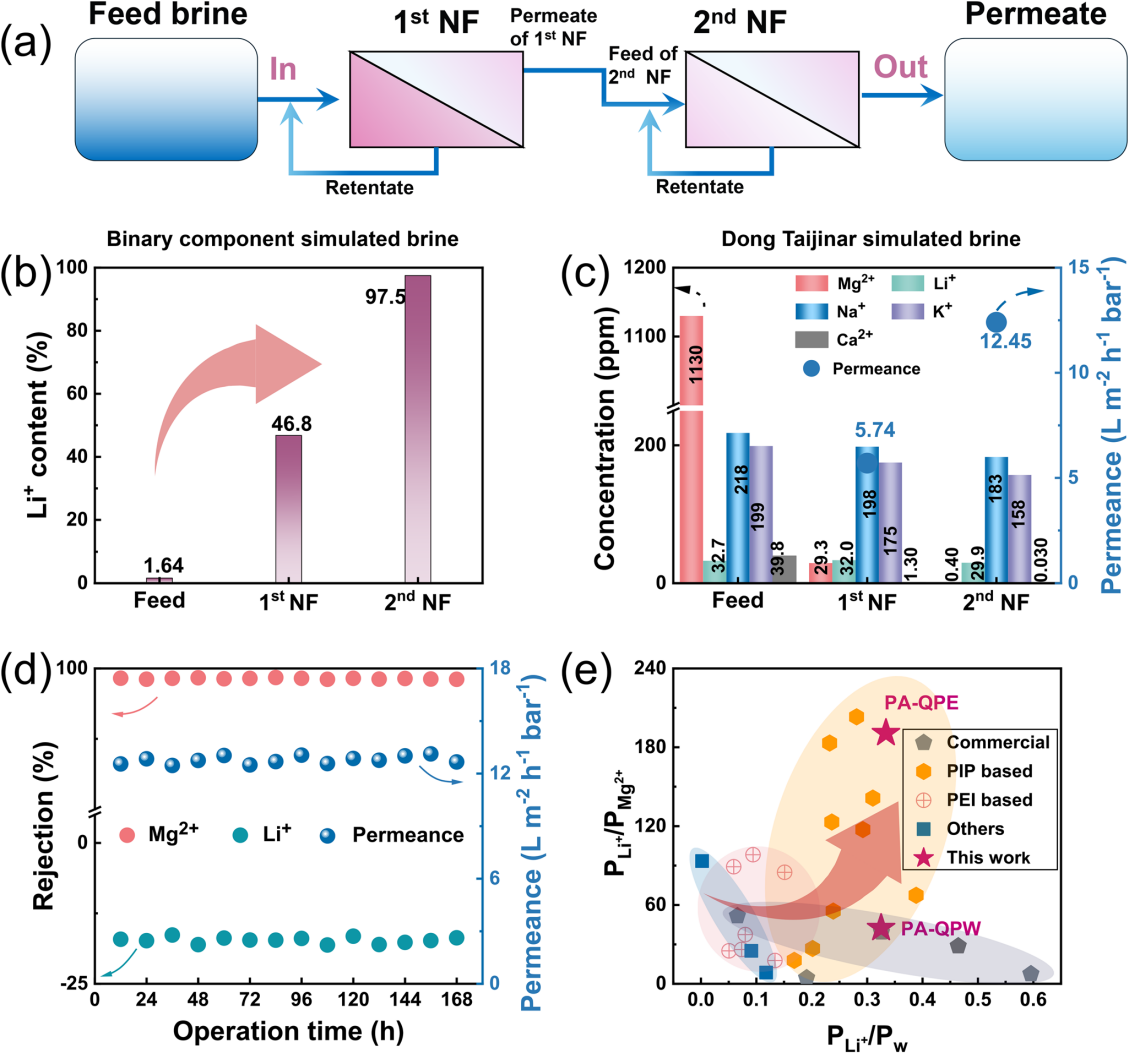
Co‐ion separation performance and application potential of the PA‐QPE membrane. (a) Schematic diagram of the two‐stage NF designed for lithium enrichment; (b) Li^+^ content enrichment in permeate and water flux after two‐stage NF (total salt concentration:10 000 ppm, Mg^2+^/Li^+^ mass ratio: 60:1); (c)Two‐stage nanofiltration performance for simulated salt lake brine (Feed: 183 ppm LiCl, 4700 ppm MgCl_2_, 609 ppm NaCl, 457 ppm KCl and 124 ppm CaCl_2_) (d) Long‐term performance stability (total salt concentration 2000 ppm, Mg^2+^/Li^+^ mass ratio: 20:1); (e) Comparison of Li^+^ purity and recovery between PA‐QPE and reported membranes.

Demonstrating the practical applicability of PA‐QPE in challenging lithium recovery scenarios, a two‐stage NF process was designed using its high ion selectivity to achieve simultaneous Li^+^ enrichment and Mg^2+^ rejection under extreme feed conditions (Figure 6a). As shown in Figure 6b and Figure S31, In a binary MgCl_2_/LiCl system with an extreme Mg^2+^/Li^+^ mass ratio of 60:1 and a total salt concentration of 10 000 ppm, sequential NF process increased the Li^+^ concentration in the permeate by nearly 60‐fold compared to the feed, while reducing Mg^2+^/Li^+^ mass ratio from 60:1  to only 0.026:1. This pronounced selectivity is attributed to the combined effects of sub‐nanometer pore size confinement, mildly positive surface potential, and the distinct hydration and dehydration energetics of Li^+^ and Mg^2+^. To further evaluate performance in a more realistic multi‐ion environment, a simulated Dong Taijinar Salt Lake brine (183 ppm LiCl, 4700 ppm MgCl_2_, 609 ppm NaCl, 457 ppm KCl, and 124 ppm CaCl_2_) was processed using the same two‐stage NF process. As shown in Figure 6c, the PA‐QPE membrane maintains superior selectivity despite the high concentration of competing ions, and after two‐stage nanofiltration, the Mg^2+^ concentration dropped to 0.40 ppm while maintaining 29.9 ppm Li^+^. Importantly, the membrane maintained both separation precision and flux stability across the two stages, underscoring its potential for real‐world lithium recovery from brines with high salt loading and severe co‐ion interference.

In addition, the designed PA‐QPE membrane also showed good long‐term operational stability over 168 hours and maintained above 12 L·m^−2^·h^−1^·bar^−1^ permeance, as well as Mg^2^^+^ rejection above 97% throughout the entire testing period with no discernible decline (Figure 6d). For a systematic evaluation of membrane performance in Li^+^/Mg^2+^ co‐ion systems, two key descriptors derived from the solution‐diffusion‐electromigration (SDEM) model were adopted: the Li^+^/Mg^2+^ permeation flux ratio (P_Li_^+^/P_Mg_^2+^) reflects lithium purity, while the Li^+^/water flux ratio (P_Li_^+^/P_w_) indicates lithium recovery efficiency [44, 45, 46]. As presented in Figure 6e and Table S4, commercial nanofiltration membranes cluster along a well‐defined trade‐off curve, where increased Li^+^ throughput typically compromises Mg^2+^ rejection, thereby limiting overall selectivity. In contrast, the PA‐QPE membrane developed in this study clearly breaks this trend, residing in the upper‐right quadrant of the performance plot and achieving both high lithium purity and recovery. This superior performance arises from its finely engineered nanostructure, characterized by a compact sub‐nanometer pore network and a mildly positive surface potential, which collectively promote selective Li^+^ transport while effectively excluding Mg^2^^+^ through the combined effects of steric hindrance and Donnan exclusion.

## 3 Conclusion

In conclusion, a reconstructed PA membrane for precise ion separation was successfully designed through a two‐stage interfacial polymerization strategy, which achieved the structural and electrostatic engineering manipulation towards the PA nanolayer. Two key issues for reconstructing the PA layer were systematically decoupled and investigated: (i) stable initial PA layer formation dominated by support, and (ii) high active sites retention for the two‐stage reaction. The physicochemical properties of the support strongly influence PIP adsorption and diffusion, thereby determining the density and stability of the primary PA layer. Meanwhile, carefully selecting non‐aqueous solvent can effectively suppress acyl chloride hydrolysis to provide abundant reaction sites and prevent excessive swelling, resulting in a compact and stable two‐stage selective layer. Importantly, the designed and synthesized bidentate quaternary ammonium monomer enabled sub‐nanometer pore confinement and imparted a mild positive surface charge, facilitating steric and electrostatic discrimination between Li^+^ and Mg^2+^.

The optimized PA‐QPE membrane exhibited outstanding Li^+^/Mg^2^^+^ selectivity with separation factors across a wide range of operational conditions and demonstrated stable performance under high Mg^2+^/Li^+^ ratios and total salt concentrations up to 6000 ppm. Two‐stage integrated nanofiltration process equipped with PA‐QPE achieved nearly 60‐fold lithium enrichment while reducing Mg^2+^/Li^+^ ratio to below 0.03, validating its practical applicability in complex brine matrices. Overall, this work establishes a scalable platform for fabricating high‐performance nanofiltration membranes capable of achieving simultaneous lithium enrichment and magnesium rejection under chemically complex, high‐salinity conditions.

## 4 Experimental Section

### 4.1 Synthesis of QP Monomer

As illustrated in Figure 1b, the quaternary ammonium monomer (QP) was synthesized via a nucleophilic substitution reaction. Pyrazine (4.16 g, 50 mmol) was dissolved in 80 mL of deionized water in a 250 mL three‐neck round‐bottom flask under continuous magnetic stirring. Subsequently, 3‐bromopropylamine hydrobromide (18.3 g, 110 mmol) was added in a single portion, and the mixture was refluxed at 80°C for 24 h under ambient pressure. Upon completion of the reaction, the resulting light‐yellow solution was concentrated under reduced pressure to approximately 40 mL and cooled in an ice bath to 0°C. Pre‐chilled anhydrous acetonitrile was then added dropwise until complete precipitation occurred. The suspension was stirred briefly and maintained at 0°C for an additional hour to ensure full crystallization. The resulting solid was collected by vacuum filtration and sequentially washed with cold acetonitrile (2 × 20 mL) and cold anhydrous ethanol (2 × 15 mL) to remove residual impurities. Finally, the product was dried under vacuum at 50°C for 12 h to yield the QP monomer as a pale‐yellow solid.

### 4.2 Preparation of PA and PA‐QP Membranes

As shown in Figure 1a, nanofiltration membranes were prepared using a two‐stage IP strategy. Initially, porous support membranes were thoroughly rinsed and equilibrated in deionized (DI) water to remove surface impurities. The porous supports were immersed in a 1 wt.% PIP aqueous solution for 2 min to allow amine adsorption, followed by brief air drying at room temperature. Subsequently, a 0.1 wt./v% solution of TMC in n‐hexane was cast onto the surface for 60 s to initiate the interfacial reaction and form the PA selective layer. To facilitate the second‐stage IP reaction, the freshly prepared PA membranes were immersed in a functional QP monomer solution for 10 min without any intermediate thermal curing. During this stage, the QP concentration varied between 0 and 3 wt.%, utilizing deionized water, ethanol, or DMSO as the solvent. Finally, the membranes were air‐dried and subjected to post‐curing at 70°C for 5 min to enhance structural stability. The membranes were systematically named according to the solvent employed; for example, the sample modified in an ethanol solution was designated as PA‐QPE, while the unmodified membrane was denoted as PA. To investigate the effect of different monomers, the freshly prepared PA membrane was immersed in synthesized AP and BP ethanol solutions (2 wt/v.%) under conditions similar to those used for QP during the second‐stage interfacial polymerization. The resultant membranes were denoted as PA‐APE and PA‐BPE, respectively.

## Conflicts of Interest

The authors declare no conflicts of interest.

## Supporting information




**Supporting File**: advs74368‐sup‐0001‐SuppMat.pdf.

## Data Availability

The data that support the findings of this study are available in the supplementary material of this article.

## References

[1] C. Liu , Y. Li , D. Lin , et al., “Lithium Extraction From Seawater Through Pulsed Electrochemical Intercalation,” Joule 4 (2020): 1459–1469, 10.1016/j.joule.2020.05.017.

[2] S. Yang , Y. Wang , H. Pan , P. He , and H. Zhou , “Lithium Extraction From Low‐Quality Brines,” Nature 636 (2024): 309–321, 10.1038/s41586-024-08117-1. 39663488

[3] T. Zhang , W. Zheng , Q. Wang , Z. Wu , and Z. Wang , “Designed Strategies of Nanofiltration Technology for Mg^2+^/Li^+^ Separation From Salt‐Lake Brine: A Comprehensive Review,” Desalination 546 (2023): 116205, 10.1016/j.desal.2022.116205.

[4] D. Qi , S. Zheng , D. Jin , et al., “A Novel Imprinted Porous Liquid for Lithium Extraction,” AIChE Journal 70 (2024): 18603, 10.1002/aic.18603.

[5] M. Yong , M. Tang , L. Sun , et al., “Sustainable Lithium Extraction and Magnesium Hydroxide Co‐Production From Salt‐Lake Brines,” Nature Sustainability 7 (2024): 1662–1671, 10.1038/s41893-024-01435-2.

[6] K. Liu , Y. Xia , X. Chen , et al., “Bilayer Crown Ether‐Engineered Nanofiltration Membranes With Dual Li^+^ Transport Channels for Ultra‐High Mg^2+^/Li^+^ Separation From Saline Lake Brines,” Water Research 284 (2025): 123924, 10.1016/j.watres.2025.123924. 40482494

[7] X. Wen , P. Ma , C. Zhu , Q. He , and X. Deng , “Preliminary Study on Recovering Lithium Chloride From Lithium‐Containing Waters by Nanofiltration,” Separation and Purification Technology 49 (2006): 230–236, 10.1016/j.seppur.2005.10.004.

[8] B. Tansel , “Significance of Thermodynamic and Physical Characteristics on Permeation of Ions During Membrane Separation: Hydrated Radius, Hydration Free Energy and Viscous Effects,” Separation and Purification Technology 86 (2012): 119–126, 10.1016/j.seppur.2011.10.033.

[9] Y. Boroumand , S. Abrishami , and A. Razmjou , “Lithium Recovery From Brines,” Nature Sustainability 7 (2024): 1550–1551, 10.1038/s41893-024-01451-2.

[10] S. H. Park , J. H. Kim , S. J. Moon , et al., “Lithium Recovery From Artificial Brine Using Energy‐Efficient Membrane Distillation and Nanofiltration,” Journal of Membrane Science 598 (2020): 117683, 10.1016/j.memsci.2019.117683.

[11] V. Freger and G. Z. Ramon , “Polyamide Desalination Membranes: Formation, Structure, and Properties,” Progress in Polymer Science 122 (2021): 101451, 10.1016/j.progpolymsci.2021.101451.

[12] F. Zhang , J. Fan , and S. Wang , “Interfacial Polymerization: From Chemistry to Functional Materials,” Angewandte Chemie International Edition 59 (2020): 21840–21856, 10.1002/anie.201916473. 32091148

[13] H. Y. Peng , S. K. Lau , and W. F. Yong , “Recent Advances of Thin Film Composite Nanofiltration Membranes for Mg^2+^/Li^+^ Separation,” Advanced Membranes 4 (2024): 100093, 10.1016/j.advmem.2024.100093.

[14] S. Fang , K. Guan , S. Zhou , et al., “Ternary‐Coordination‐Regulated Polyamide Nanofiltration Membranes for Li^+^/Mg^2+^ Separation,” Desalination 581 (2024): 117577, 10.1016/j.desal.2024.117577.

[15] H. Li , Y. Li , M. Li , Y. Jin , G. Kang , and Y. Cao , “Improving Mg^2+^/Li^+^ Separation Performance of Polyamide Nanofiltration Membrane by Swelling‐Embedding‐Shrinking Strategy,” Journal of Membrane Science 669 (2023): 121321, 10.1016/j.memsci.2022.121321.

[16] H. Peng , K. Yu , X. Liu , J. Li , X. Hu , and Q. Zhao , “Quaternization‐Spiro Design of Chlorine‐Resistant and High‐Permeance Lithium Separation Membranes,” Nature Communications 14 (2023): 5483, 10.1038/s41467-023-41169-x. PMC10482931 37673942

[17] S. Zhao , L. Dai , Z. Mai , et al., “Nanoconfinement Engineering of Covalent Organic Frameworks in Polyamide Membranes for High‐Perselectivity Li^+^/Mg^2+^ Separation,” Advanced Science 12 (2025): 2500255, 10.1002/advs.202500255. 40171948 PMC12199429

[18] L. Liu , Y. Liu , Y. Wan , W. Song , H. Lu , and J. Luo , “Ionic Liquid‐Induced Deep Grafting of Polyelectrolyte to Reform Polyamide Layer for Antifouling Nanofiltration Membrane,” AIChE Journal 69 (2023): 18204, 10.1002/aic.18204.

[19] M.‐J. Tang , M.‐L. Liu , L. Li , et al., “Solvation‐Amination‐Synergy that Neutralizes Interfacially Polymerized Membranes for Ultrahigh Selective Nanofiltration,” AIChE Journal 68 (2022): 17602, 10.1002/aic.17602.

[20] Z. Wang , S. Yuan , D. Wang , N. Zhang , Y. Shen , and Z. Wang , “N‐Oxide Zwitterionic‐Based Antifouling Loose Nanofiltration Membranes with Superior Water Permeance and Effective Dye/Salt Separation,” Environmental Science & Technology 59 (2025): 5856–5865, 10.1021/acs.est.5c00916. 40068006

[21] J. Zhang , T. Huhe , Z. Zhou , D.‐D. Shao , X. Meng , and Q. Wang , “Enhancement of Lignin‐Modified Nanofiltration Membranes Performance via Acyl Chloride Crosslinking Post‐Treatment,” Separation and Purification Technology 377 (2025): 134255, 10.1016/j.seppur.2025.134255.

[22] S. Guo , X. Chen , Y. Wan , S. Feng , and J. Luo , “Custom‐Tailoring Loose Nanofiltration Membrane for Precise Biomolecule Fractionation: New Insight Into Post‐Treatment Mechanisms,” ACS Applied Materials & Interfaces 12 (2020): 13327–13337, 10.1021/acsami.0c00259. 32109041

[23] D. Lu , T. Ma , S. Lin , et al., “Constructing a Selective Blocked‐Nanolayer on Nanofiltration Membrane via Surface‐Charge Inversion for Promoting Li^+^ Permselectivity Over Mg^2+^ ,” Journal of Membrane Science 635 (2021): 119504, 10.1016/j.memsci.2021.119504.

[24] Y. Fan , H. Tian , K. Wang , et al., “High Positively Charged Composite Nanofiltration Membranes Modified by a Novel Bis‐Quaternary Ammonium Monomer for Li^+^ Extraction From High Mg^2+^/Li^+^ Ratio Salt Lakes,” Separation and Purification Technology 358 (2025): 130276, 10.1016/j.seppur.2024.130276.

[25] T. Gu , R. Zhang , S. Zhang , et al., “Quaternary Ammonium Engineered Polyamide Membrane With High Positive Charge Density for Efficient Li^+^/Mg^2+^ Separation,” Journal of Membrane Science 659 (2022): 120802, 10.1016/j.memsci.2022.120802.

[26] A. K. Ghosh and E. M. V. Hoek , “Impacts of Support Membrane Structure and Chemistry on Polyamide–Polysulfone Interfacial Composite Membranes,” Journal of Membrane Science 336 (2009): 140–148, 10.1016/j.memsci.2009.03.024.

[27] Y. J. Lim , K. Goh , G. S. Lai , Y. Zhao , J. Torres , and R. Wang , “Unraveling the Role of Support Membrane Chemistry and Pore Properties on the Formation of Thin‐Film Composite Polyamide Membranes,” Journal of Membrane Science 640 (2021): 119805, 10.1016/j.memsci.2021.119805.

[28] X. Li , Q. Li , W. Fang , R. Wang , and W. B. Krantz , “Effects of the Support on the Characteristics and Permselectivity of Thin Film Composite Membranes,” Journal of Membrane Science 580 (2019): 12–23, 10.1016/j.memsci.2019.03.003.

[29] S. Zhao , W. Cui , Q. Shen , et al., “Porous Organic Polymer Interlayers Modulated Nanofiltration Membranes for Ultra‐Permselective Li^+^/Mg^2+^ Separation,” Journal of Membrane Science 690 (2024): 122207, 10.1016/j.memsci.2023.122207.

[30] A. Kv , M. R. Puhan , D. B. Vasave , T. Gohil , S. Karan , and B. Sutariya , “Are Hansen Solubility Parameters Relevant in Predicting the Post‐Treatment Effect on Polyamide‐Based TFC Membranes?,” Environmental Science and Pollution Research 31 (2024): 21157–21171, 10.1007/s11356-024-32520-w. 38388971

[31] M. G. Shin , W. Choi , J. Huh , et al., “Solvent Transport Model for Polyamide Nanofilm Membranes Based on Accurate Hansen Solubility Parameters,” Journal of Membrane Science 674 (2023): 121505, 10.1016/j.memsci.2023.121505.

[32] M. R. Puhan , B. Sutariya , and S. Karan , “Revisiting the Alkali Hydrolysis of Polyamide Nanofiltration Membranes,” Journal of Membrane Science 661 (2022): 120887, 10.1016/j.memsci.2022.120887.

[33] F. Soyekwo , C. Liu , X. Mao , and X. Shi , “Poly(bis(1‐methylpiperazin‐1‐ium‐amide) Nanofilm Composite Membrane with Nanochannel‑Enabled Microporous Structure and Enhanced Steric Hindrance for Magnesium/Lithium Separation,” Advanced Functional Materials 35 (2024): 2412463, 10.1002/adfm.202412463.

[34] V. Freger , “Nanoscale Heterogeneity of Polyamide Membranes Formed by Interfacial Polymerization,” Langmuir 19 (2003): 4791–4797, 10.1021/la020920q.

[35] H. Peng , Q. Tang , S. Tang , J. Gong , and Q. Zhao , “Surface Modified Polyamide Nanofiltration Membranes With High Permeability and Stability,” Journal of Membrane Science 592 (2019): 117386, 10.1016/j.memsci.2019.117386.

[36] S. Jeon , H. Kim , J. Choi , J. F. Kim , H. B. Park , and J.‐H. Lee , “Extreme pH‐Resistant, Highly Cation‐Selective Poly(Quaternary Ammonium) Membranes Fabricated via Menshutkin Reaction‐Based Interfacial Polymerization,” Advanced Functional Materials 33 (2023): 2300183, 10.1002/adfm.202300183.

[37] L. Long , C. Wu , S. Shao , Z. Yang , P. Sarkar , and C. Y. Tang , “Assessment of Permeance and Selectivity of Thin‐Film Composite Polyamide Membranes for Diverse Applications,” Nature Water 3 (2025): 668–682.

[38] H. Xu , D. Lan , J. Wang , et al., “Nanofiltration Membranes for Mg^2+^/Li^+^ Separation: Separation Mechanisms, Mass Transfer Models and Current Research Advances,” Separation and Purification Technology 370 (2025): 133206, 10.1016/j.seppur.2025.133206.

[39] L. Liu , S. Lin , X. Xu , Y. Wan , W. Song , and J. Luo , “Preference of Negatively Charged Membranes in Magnesium and Lithium Separation by Nanofiltration,” Nature Communications 16 (2025): 5731, 10.1038/s41467-025-61336-6. PMC12216317 40595735

[40] Q. Peng , R. Wang , Z. Zhao , et al., “Extreme Li‐Mg Selectivity via Precise Ion Size Differentiation of Polyamide Membrane,” Nature Communications 15 (2024): 2505, 10.1038/s41467-024-46887-4. PMC10954764 38509082

[41] L. Liu , S. Lin , X. Xu , Y. Wan , W. Song , and J. Luo , “Preference of Negatively Charged Membranes in Magnesium and Lithium Separation by Nanofiltration,” Nature Communications 16 (2025): 5731, 10.1038/s41467-025-61336-6. PMC12216317 40595735

[42] F. Yang , M. Yong , Z. Li , Z. Yang , and X. Zhang , “Breaking the Trade‐Off Between Lithium Purity and Lithium Recovery: A Comprehensive Mathematical Modeling Based on Membrane Structure‐Property‐Performance Relationships,” Water Research 281 (2025): 123678, 10.1016/j.watres.2025.123678. 40280005

[43] G. Xuerui , L. Ping , Q. Yuan , B. Chengling , G. Zhengyang , and Y. Shuili , “Negative Rejection Phenomenon in the Mixed Salt Nanofiltration: Law and Mechanism,” Desalination 583 (2024): 117667, 10.1016/j.desal.2024.117667.

[44] B. Yuan , Y. Zhang , P. Qi , et al., “Self‐Assembled Dendrimer Polyamide Nanofilms With Enhanced Effective Pore Area for Ion Separation,” Nature Communications 15 (2024): 471, 10.1038/s41467-023-44530-2. PMC10784486 38212318

[45] B. Yuan , M. Wang , M. Wu , et al., “Asymmetric Polyamide Nanofilm Boosted by Protonated Dendrimer Porous Intermediate Layer for Li^+^/Mg^2+^ Separation,” Journal of Membrane Science 701 (2024): 122743, 10.1016/j.memsci.2024.122743.

[46] R. Wang , R. He , T. He , M. Elimelech , and S. Lin , “Performance Metrics for Nanofiltration‐Based Selective Separation for Resource Extraction and Recovery,” Nature Water 1 (2023): 291–300, 10.1038/s44221-023-00037-0.

