# Supplementary material for: Reconstructed Polyamide Nanolayers via Two‐Stage Interfacial Polymerization Engineering for Precise Ion Sieving
Source: Adv Sci (Weinh). 2026 Feb 17;13(23):e74368. doi: 10.1002/advs.74368 (PMC13104069; doi:10.1002/advs.74368)
Supplement: Supplementary file 1 — Supporting File: advs74368‐sup‐0001‐SuppMat.pdf. [file ADVS-13-e74368-s001.pdf]

Supporting Information

Reconstructed Polyamide Nanolayers via Two-Stage Interfacial Polymerization for Precise Ion Sieving

Shuzhen Zhao [a,b], Liheng Dai *[a], Feidong Yang [a,b], Bowen Li [a,b], Pengfei Zhang [a], Yanyan Liu [a], Mengyang Hu [a], Kecheng Guan [a], Ryosuke Takagi [a], Hideto Matsuyama *[a,b]

[a] *Research Center for Membrane and Film Technology, Kobe University, 1-1 Rokkodaicho, Nada, Kobe, 657-8501, Japan*

[b] *Department of Chemical Science and Engineering, Kobe University, 1-1 Rokkodaicho, Nada, Kobe, 657-8501, Japan*

**Corresponding author, email:* matuyama@kobe-u.ac.jp; *lhengdai@people.kobe-u.ac.jp;*

Experimental

Materials

Commercial PSF (US-050) and PES (ES-050) membranes were acquired from RisingSun Membrane Technology (Beijing) Co., Ltd and used as the substrates for the IP process. Aliphatic polyketone (PK, *Mw* ≈ 2,000,000 g·mol⁻1) powder was supplied by Asahi Kasei Corp. (Tokyo, Japan). All analytical-grade reagents and solvents, including acetonitrile, methanol, acetone, n-hexane, ethanol (99.5%), and 1,3,5-benzenetricarbonyl chloride (TMC), were purchased from FUJIFILM Business Innovation Co. lithium chloride (LiCl), magnesium chloride hexahydrate ($MgCl_2·6H_2O$), magnesium sulfate ($MgSO_4$), sodium chloride (NaCl), potassium chloride (KCl), calcium chloride ($CaCl_2$), copper(II) chloride ($CuCl_2$), cobalt(II) chloride ($CoCl_2$), barium chloride ($BaCl_2$), sodium sulfate (Na_2SO_4), and nickel(II) chloride hexahydrate ($NiCl_2·6H_2O$). All were obtained from FUJIFILM Business Innovation unless otherwise specified. Organic reagents including resorcinol, pyrazine, and 3-bromopropylamine hydrobromide were obtained from Tokyo Chemical Industry Co., Ltd. Piperazine (PIP, anhydrous), along with neutral solutes employed for molecular weight cutoff evaluation, glycerol (*Mw* = 92.1 Da), D-(+)-glucose (*Mw* = 180.2 Da), sucrose (*Mw* = 342.3 Da), and D-(+)-raffinose pentahydrate (*Mw* = 594.5 Da), were purchased from Sigma-Aldrich and used without further purification.

Preparation of PK substrates

Based on previously established research in our group[1,2], the PK porous support

membrane was fabricated via a conventional non-solvent induced phase separation (NIPS) technique using a polyester nonwoven fabric as the backing layer. To prepare the casting solution, 14 wt% PK powder was dispersed in a resorcinol/deionized water mixture (65:35, w/w%) and stirred at 80 °C for 5 h to ensure complete dissolution and homogenization. The solution was then degassed under ambient conditions overnight to remove trapped air bubbles. The degassed solution was cast onto the polyester substrate using a 400 μm casting knife and immediately immersed in a coagulation bath composed of methanol and deionized water (35:65, w/w%) for 20 min to induce phase separation and form the nascent membrane. Following coagulation, the membrane was sequentially washed with acetone and n-hexane (20 min each) to extract residual solvents and enhance pore cleanliness. The resulting PK porous membranes were air-dried and stored at room temperature under dry conditions until further use.

Characterization

The molecular structure of the synthesized quaternary ammonium monomer was verified via proton nuclear magnetic resonance (^{1}H NMR) spectroscopy using a Bruker AV400 spectrometer. To investigate the chemical composition and surface functionalities of the prepared membranes, attenuated total reflectance Fourier-transform infrared (ATR-FTIR, PerkinElmer) spectroscopy and X-ray photoelectron spectroscopy (XPS, JPS-9010MC, JEOL) were employed. Surface wettability was quantified by measuring static water contact angles (WCA) with a Drop Master 300 goniometer (Kyowa, Japan), while electrokinetic properties were assessed through zeta potential analysis using a solid

surface analyzer (Anton Paar, Australia). The surface morphology, cross-sectional architecture, and selective layer thickness were examined by field-emission scanning electron microscopy (FESEM, Sigma 500, ZEISS). Topographical features and surface roughness were further characterized by atomic force microscopy (AFM, Bruker Dimension Icon). Meanwhile, the concentrations of ionic species in brine and permeate solutions were precisely measured using inductively coupled plasma mass spectrometry (ICP-MS, Shimadzu).

The pore size characteristics and molecular weight cut-off (MWCO) of the membranes were evaluated based on the neutral solute transport method. Aqueous solutions containing four polysaccharides, glycerol, glucose, sucrose, and raffinose, as well as five dextrans with varying molecular weights (200 ppm each), were employed as model neutral solutes. These solutes were selected to cover a broad molecular size range for a comprehensive assessment of both the porous ultrafiltration supports and the PA separation layers. Solute rejection tests were performed individually, and the rejection ratios were quantified using a total organic carbon analyzer (TOC-V, Shimadzu). The resulting data were used to calculate the apparent MWCO values and to derive the membrane pore size distribution curves based on equation 1.

$$\frac{dR(r_p)}{dr_p} = \frac{1}{r_p\, ln\sigma_p\sqrt{2\pi}} exp\left[-\frac{\left(lnr_p - ln\mu_p\right)^2}{2\left(ln\sigma_p\right)^2}\right] \qquad (S1)$$

Here, the membrane pore size (μ_p, nm) is the same as the solute size (r_p, nm) at a rejection rate of 50%; the Stokes radius (r_s, nm) equals the solute size at a rejection rate of 84.13%, and the geometric standard (σ_p) is calculated as the ratio of ds to r_p.

The Stokes radius (r_p, nm) of these neutral organic compounds was calculated using the following equations.

For small neutral carbohydrate molecules:

$$log(r_p) = -1.4962 + 0.4654 log M_w \quad (S2)$$

For Dextrans molecules:

$$r_p = 10.44 \times 10^{-3} {M_W}^{0.587} \quad (S3)$$

Based on the above analysis, the Stokes radius of glycerol, glucose, sucrose, and raffinose were 0.26, 0.36, 0.48, and 0.58 nm, respectively.

PIP diffusion rates into n-hexane of different substrates

To evaluate the diffusion behavior of PIP monomers into n-hexane, a procedure analogous to the IP process was utilized. Initially, 25 ml of aqueous PIP solution was applied to the surface of substrates for 2 minutes. Excess solution was then removed using an air knife. The substrates were subsequently immersed in 20 ml of pure n-hexane (without TMC) for 60 seconds. After this, 1 ml of a hexane solution containing PIP monomer was carefully pipetted at the hexane-air interface. The concentration of PIP in the n-hexane phase was measured using UV–Vis absorption spectroscopy, providing a basis for calculating the diffusion rate of PIP into n-hexane[3,4].

PIP storage capacity of different substrates

The substrates with an effective surface area of 20.25 cm^2 were first immersed in 30 ml of an aqueous PIP solution (1 w/v%) for 2 minutes. Afterward, any excess liquid was removed from the substrates using an air knife. The substrates were then transferred to 40

ml of n-hexane without TMC, where they remained until the PIP monomers had fully diffused. The amount of PIP absorbed onto the substrate was quantified using UV–Vis absorption spectroscopy, with PIP showing a characteristic absorption peak near 198 nm, which corresponds to its concentration in n-hexane[5].

Separation performance test of membranes

The inorganic salt rejection (R) and permeance (J) of the NF membranes were evaluated using a cross-flow filtration system with an effective filtration area of 20.42 cm^2, and all concentration of salt solution was 2 g L^{-1}.

The water permeance (*J*), salt rejection (*R*), and single salt $LiCl/MgCl_2$ selectivity ($S_{LiCl/MgCl_2}$)were calculated by following the equations.

$$J = \frac{\Delta V}{\Delta P \times \Delta t \times S} \quad (S4)$$

$$R = \left(1 - \frac{C_p}{C_f}\right) \times 100\% \quad (S5)$$

$$S_{LiCl/MgCl_2} = \frac{1 - R_{LiCl}}{1 - R_{MgCl_2}} \quad (S6)$$

Here ΔV (L) represents the volume of permeated water over a time interval Δt (h), and ΔP is the transmembrane pressure. A transmembrane pressure of 10 bar and 2 bar was applied to evaluate the permeance of the PA-based membranes and PSF ultrafiltration supports, respectively. S (m^2) denotes the effective filtration area. All membrane samples were pre-compacted for 2 hours until a stable water permeance was achieved before data collection. The reported water permeance values were calculated as the average of three

measurements for samples prepared in different batches. Where C_p and C_f represent the concentrations of the permeate and feed solutions, respectively. The rejection rates, R_{LiCl} and R_{MgCl_2} correspond to the rejection of LiCl and $MgCl_2$ solutions, respectively.

To further investigate the separation selectivity of the membrane for mixed salt solution ($MgCl_2$/LiCl), the separation factor ($S_{Li^+/Mg^{2+}}$) was also calculated according to this Equation.

$$S_{Li^+/Mg^{2+}} = \frac{\left(C_{f\,Mg^{2+}}/C_{f\,Li^+}\right)}{\left(C_{p\,Mg^{2+}}/C_{p\,Li^+}\right)} = \frac{1 - R_{Li^+}}{1 - R_{Mg^{2+}}} \qquad (S7)$$

Here, $C_{f\,Mg^{2+}}$, $C_{f\,Li^+}$, $C_{p\,Mg^{2+}}$ and $C_{p\,Li^+}$ represent the concentrations of Mg^{2+} and Li^+ in the feed and permeate, respectively. Each membrane sample was tested at least three times, with three separate membranes used for reproducibility.

For single-component solutions containing only Mg^{2+} or Li^+, the ion concentrations were determined using an ion conductivity meter (Horiba, Japan). For the Mg^{2+}/Li^+ mixed solutions, the concentrations of Li^+ and Mg^{2+} were measured using an inductively coupled plasma optical emission spectrometer.

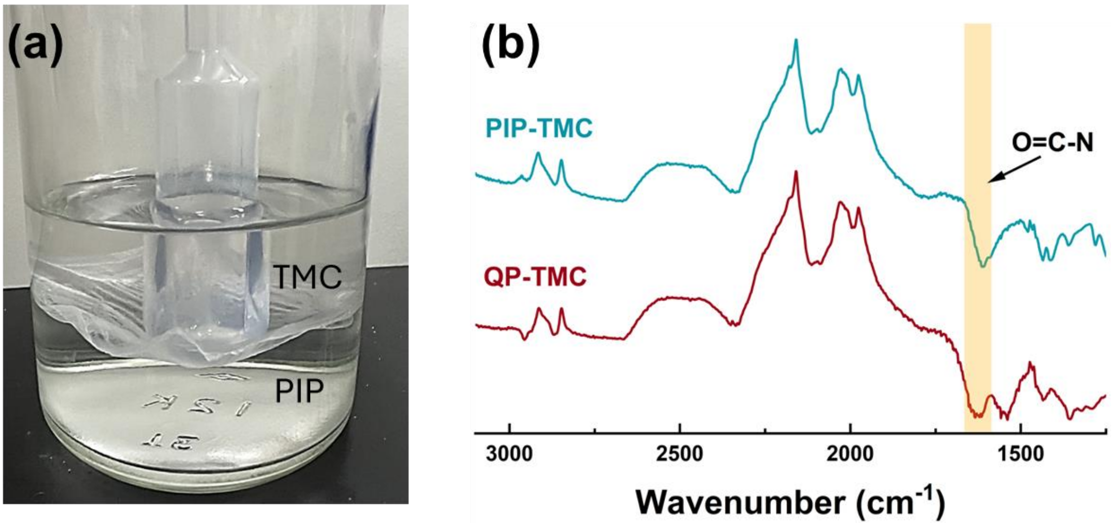

Figure S1 (a) The optical image of interfacial polymerization between PIP monomer and TMC; (b) FTIR of freestanding membrane made from QP or PIP and TMC.

The quaternary ammonium salt was synthesized by reacting pyrazine with two equivalents of 3-bromopropylamine hydrobromide in water, affording 1,4-bis(3-aminopropyl) pyrazine-1,4-diium bromide (designated as QP). The resulting ^{1}H NMR spectrum acquired in D_2O exhibits four dominant resonances, which are located at δ 8.85-8.95 ppm (4 H, position 1), 4.55-4.70 ppm (4 H, position 2), 2.60-2.80 ppm (4 H, position 3) and 2.95-3.10 ppm (4 H, position 4), confirming the successful synthesis of the QP monomer. To assess the new monomer's ability to rapidly form a film with TMC, a 1 wt% aqueous solution of the monomer was contacted with n-hexane containing 0.1 wt% TMC at 25 °C. A continuous, self-supporting film was formed at the water-organic interface within 1 min.

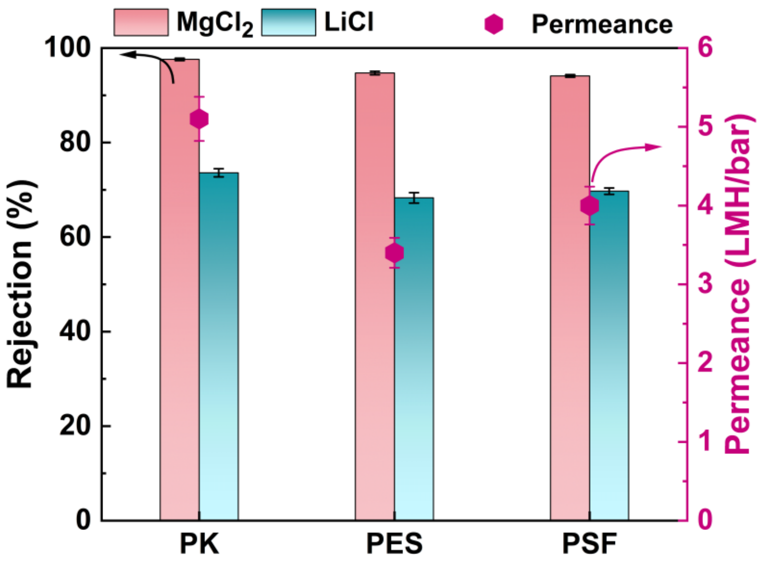

Figure S2 Separation performance of nanofiltration membranes fabricated by direct interfacial polymerization of QP and TMC on different supports

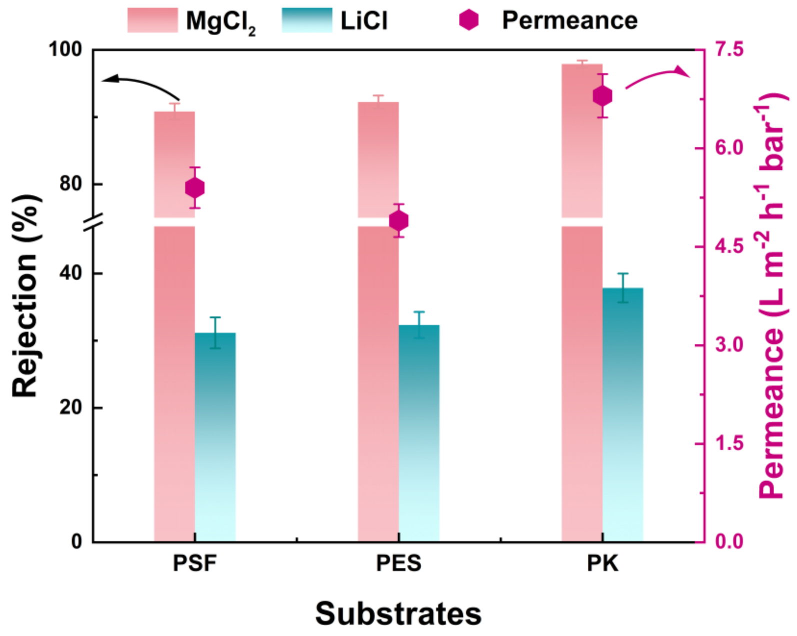

Figure S3 Separation performance of PA membranes via primary IP process on various substrates

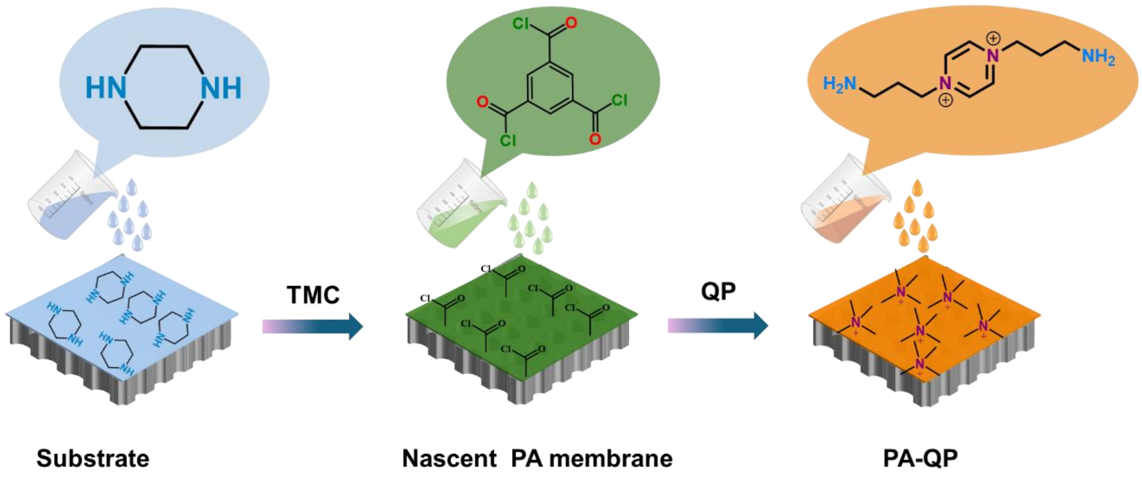

Figure S4 Diagram of QP-Based Secondary Interfacial Polymerization Strategy

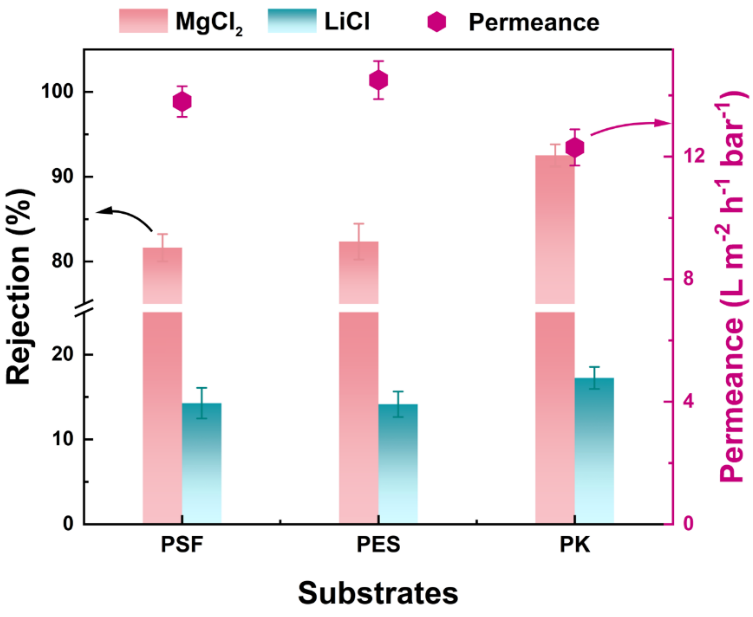

Figure S5 Separation performance of PA membranes via secondary IP process on various substrates

Table S1 The surface elemental composition and crosslinking degree of membranes.

Membranes	C (%)	O (%)	N (%)	O/N	DC (%)
**PSF-PA**	70.57	16.29	13.14	1.24	67.9
**PES-PA**	70.15	16.64	13.21	1.26	65.5
**PK-PA**	68.51	16.85	14.64	1.15	79.0

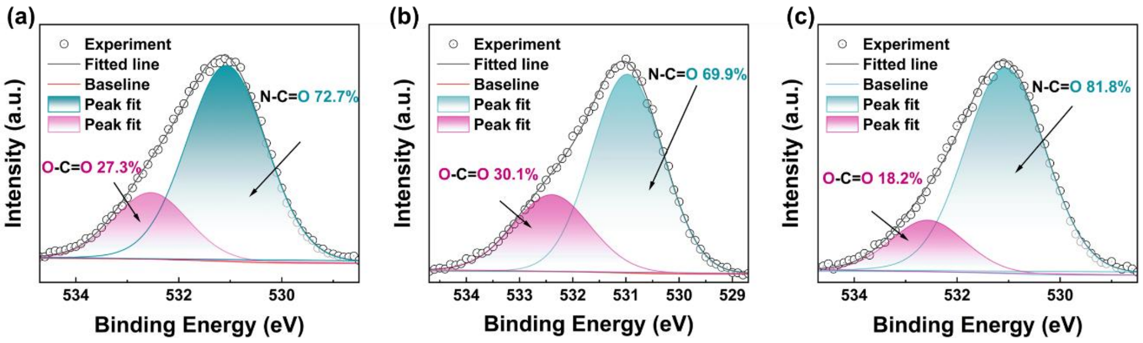

Figure S6 O 1s XPS spectra of PA membranes formed on the (a) PSF, (b) PES and (c) PK substrates.

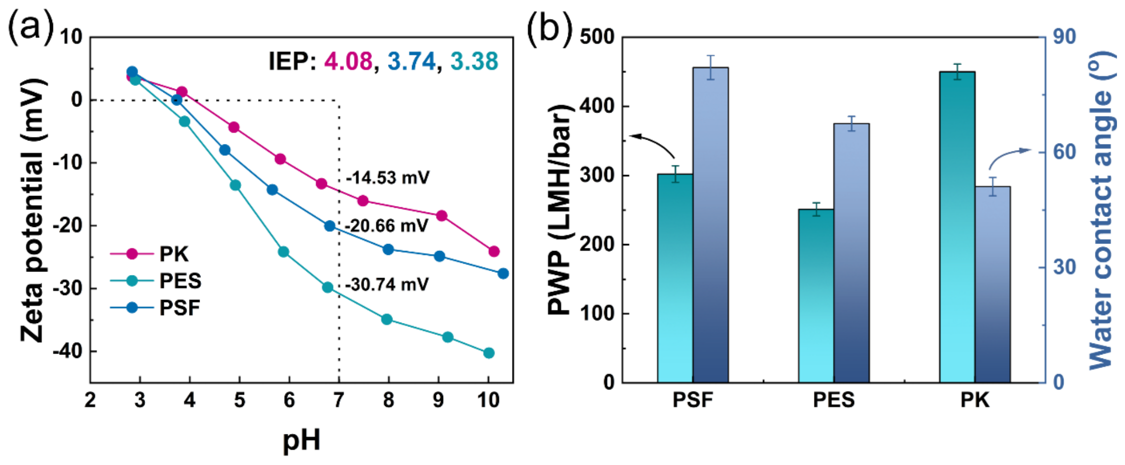

Figure S7 (a) Surface Zeta potential and (b) Pure water permeance and surface water contact angle of different substrates.

The zeta potentials of all three membranes exhibit a progressive decline with increasing pH, corresponding to an enhanced negative surface charge density. Notably, PSF displays the most pronounced decrease, whereas PK shows the most gradual variation, suggesting a relatively stable surface charge environment. This behavior correlates well with their isoelectric points (IEPs), where PK possesses the highest IEP (4.08), followed by PES (3.74) and PSF (3.38). Figure S7(b) presents the pure water permeability (PWP) and water contact angles of the membranes. PK demonstrates the highest PWP, significantly outperforming both PES and PSF, indicating excellent water transport properties. Moreover, it also has the lowest contact angle, reflecting superior hydrophilicity. In contrast, PSF exhibits the highest contact angle and the lowest PWP, suggesting poor hydrophilicity and limited water permeability.

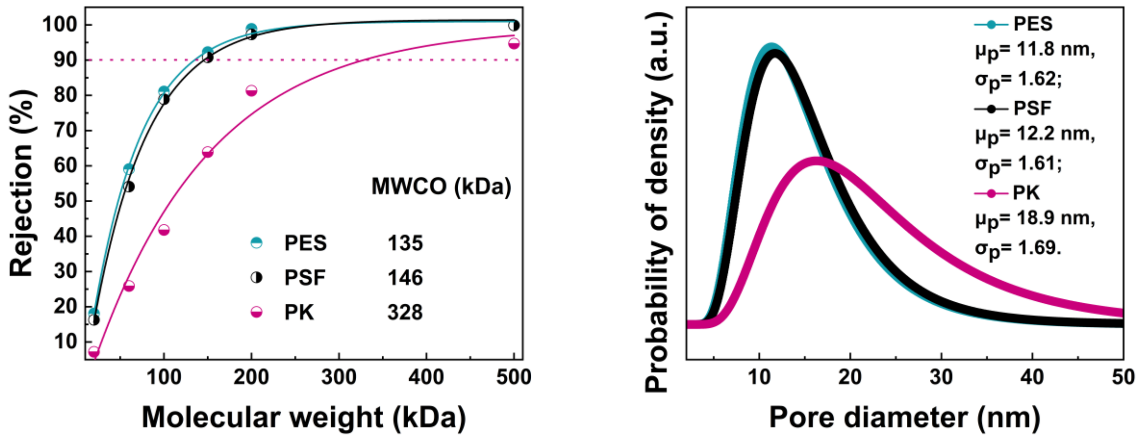

Figure S8 (a) Molecular weight cut-off and (b) pore size distribution of different substrates.

Figure S8(a) shows the rejection curves for solutes with varying molecular weights, from which the molecular weight cut-off (MWCO), defined as the molecular weight corresponding to 90% rejection, can be determined. The PK substrate exhibits a significantly higher MWCO of 328 kDa, compared to 135 kDa for PES and 146 kDa for PSF, indicating a larger pore structure. Figure S8(b) presents the pore size distribution of the three substrates. PES and PSF show relatively narrow distributions, with average pore diameters of 11.8 nm and 12.2 nm, and standard deviations (σ_p) of 1.62 and 1.61, respectively. In contrast, the PK substrate has a larger average pore diameter of 18.9 nm and a broader distribution (σ_p = 1.69), which aligns with its higher MWCO. These results suggest that the PK substrate possesses a looser and more open pore architecture, which may influence subsequent membrane fabrication.

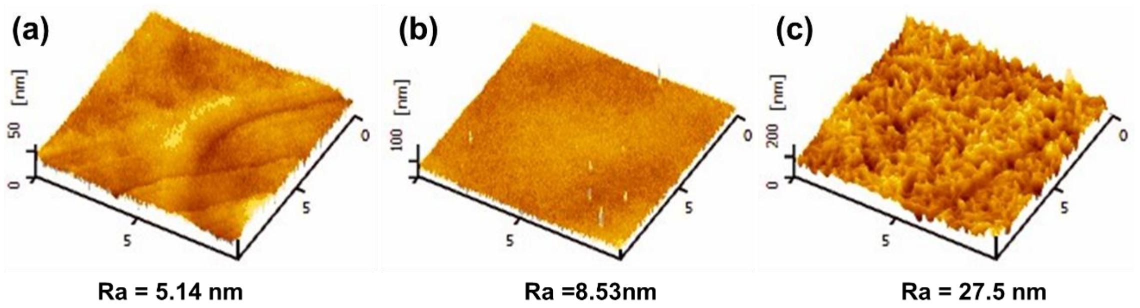

Figure S9 Surface AFM morphology and roughness of the (a) PSF, (b) PES and (c) PK substrates.

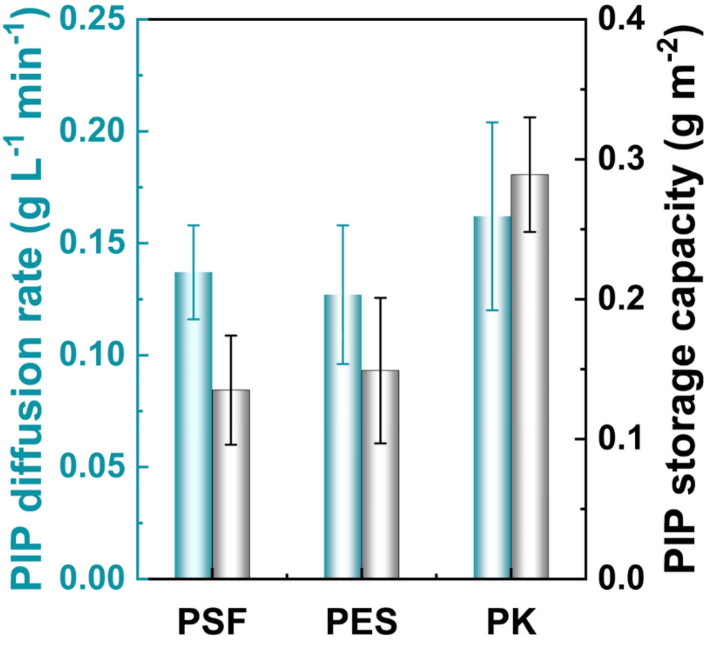

Figure S10 PIP storage capacity of different supports and its diffusion rate into the organic phase

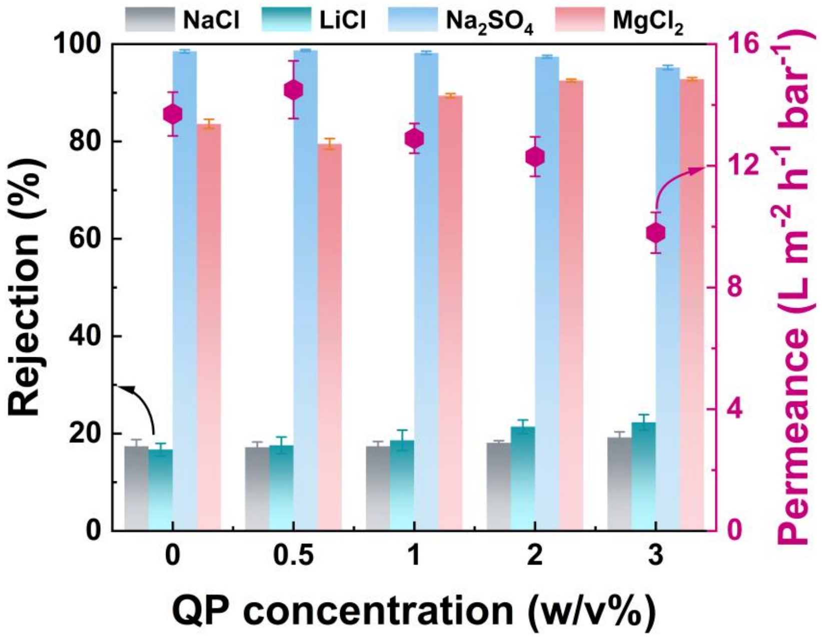

Figure S11 Optimization of QP concentration in water.

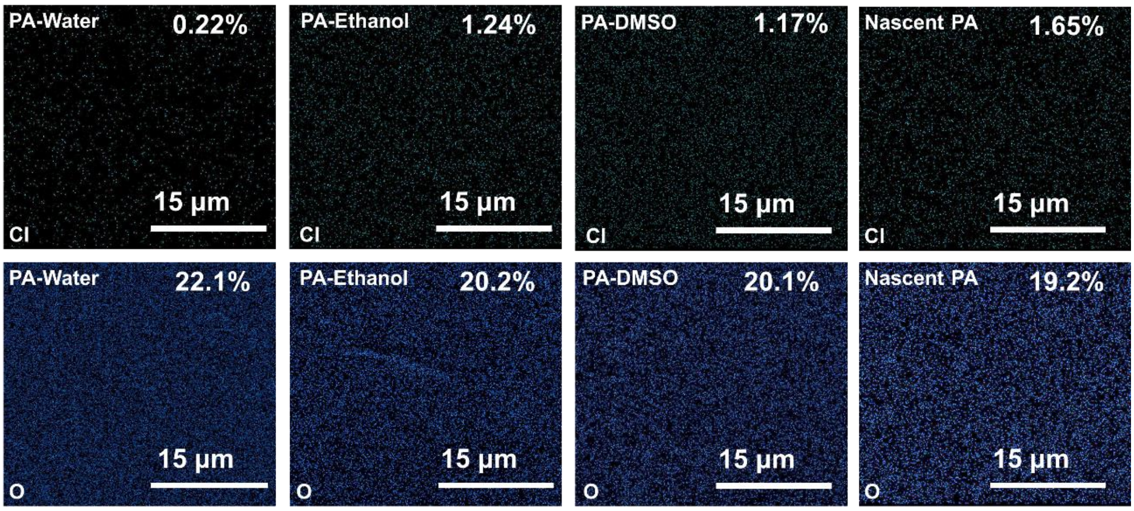

Figure S12 Elemental mapping images and quantitative surface compositions of the membranes

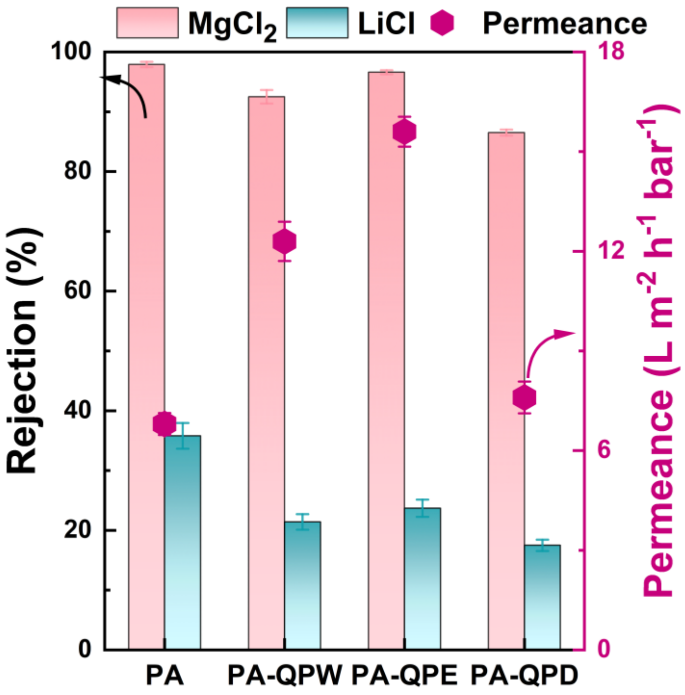

Figure S13. Separation performance of PA membranes prepared via QP-based secondary interfacial polymerization under different solvent conditions. **Note**: the membranes optimized via secondary interfacial polymerization in ethanol, DMSO and aqueous systems are hereafter referred to as PA-QPE PA-QPD and PA-QPW, respectively.

Table S2 Hansen Solubility Parameters and Distance (Ra) between Various Solvents and PA Segments

	HSPs ($MPa^{1/2}$)			
	δ_D	δ_P	δ_H	Ra
**PA layer**	20.6	12.6	9.7	--
**Water**	15.5	16.0	42.3	34.33
**Ethanol**	15.8	8.8	19.4	14.17
**DMSO**	18.4	16.4	10.2	5.84

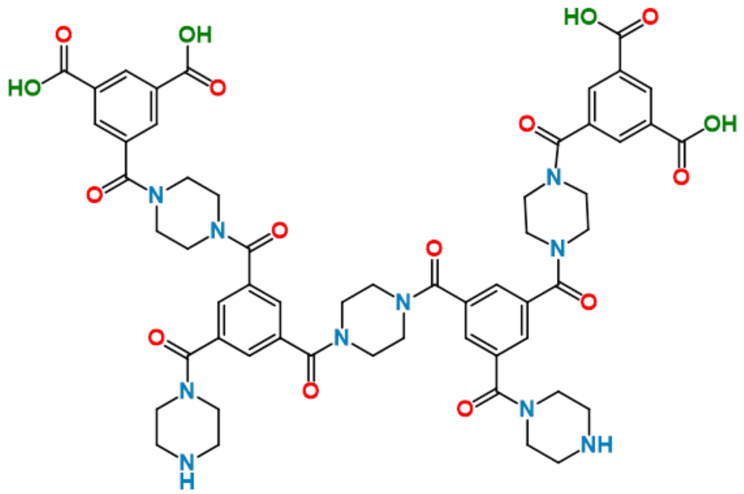

Figure S14 The PA molecular chain unit adopted for HSP computation

A representative molecular segment of the PA layer, as shown in Figure S14, was selected to characterize its chemical structure. Using the HSPiP software, the Hansen solubility parameters (HSP) of the PA segment were simulated and calculated, as presented in Figure S15. Based on the obtained HSP values, the Hansen solubility parameter distance (Ra) between various solvents and the PA layer was further calculated, and the results are summarized in Table S2.

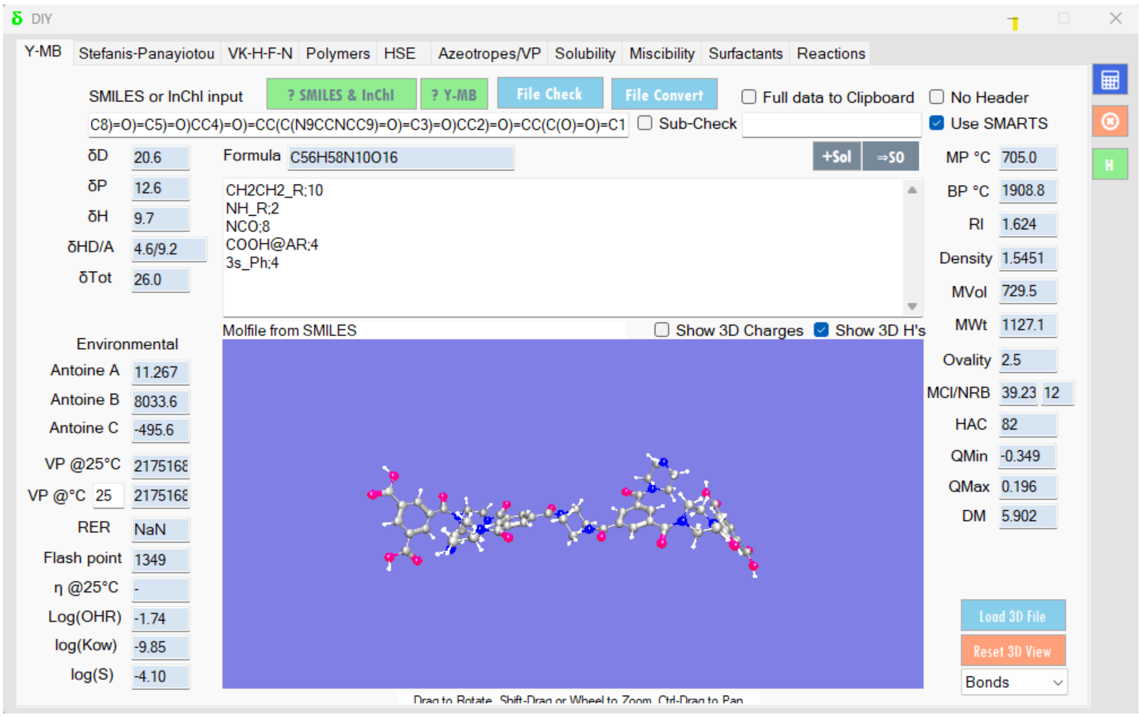

Fig. S15 Solubility parameter modeling and visualization interface in HSPiP

Hansen Solubility Parameters (HSPs) provide a widely accepted theoretical framework for understanding and predicting solubility behavior in multi-component molecular systems. Extending from Hildebrand's single-parameter approach, Charles M. Hansen proposed a three-parameter model that accounts for the distinct types of intermolecular forces: dispersion, polar, and hydrogen bonding. The original Hildebrand solubility parameter is defined based on the square root of the cohesive energy density, expressed as;

$$\delta = \sqrt{\frac{E}{V_m}} \qquad (S8)$$

Where E is the energy of vaporization and V_m is the molar volume of the substance.

However, this approach does not distinguish between different intermolecular forces. Hansen

refined the model by decomposing the total solubility parameter δ_T into three components.

$$\delta_{T^2} = \delta_{D^2} + \delta_{P^2} + \delta_{H^2} \quad (S9)$$

Here, δ_D denotes dispersion forces, δ_P corresponds to polar interactions, and δ_H captures hydrogen bonding strength.

The distance Ra between a solvent and PA layer is given by:

$$R_a = \left[4\left(\delta_{D_a} - \delta_{D_{PA}}\right)^2 + \left(\delta_{P_a} - \delta_{P_{PA}}\right)^2 + \left(\delta_{H_a} - \delta_{H_{PA}}\right)^2\right]^{\frac{1}{2}} \quad (S10)$$

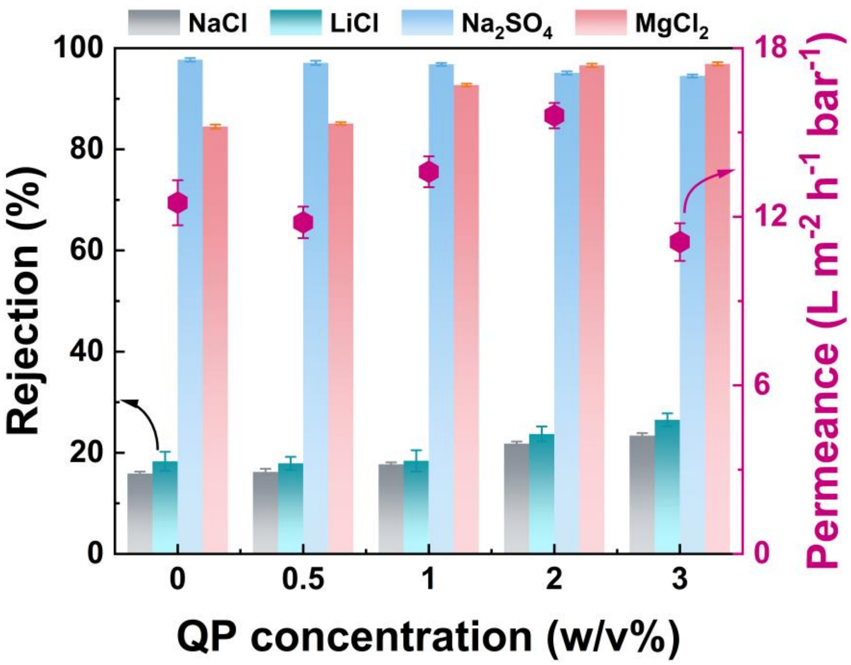

Figure S16. Optimization of QP concentration in Ethanol.

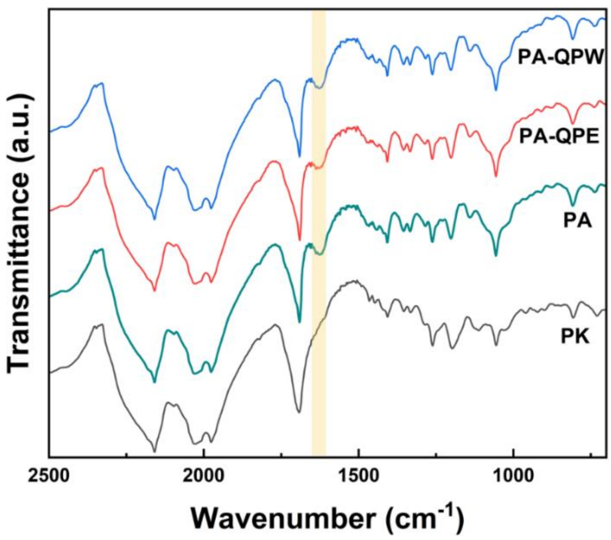

Figure S17 ATR-FTIR spectra of membranes. **Note**: the membranes optimized via secondary interfacial polymerization in ethanol and aqueous systems are hereafter referred to as PA-QPE and PA-QPW, respectively.

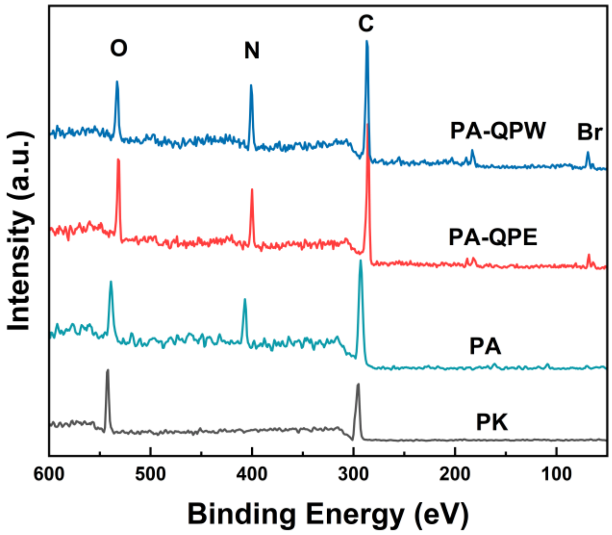

Figure S18 XPS spectra of membranes. **Note**: the membranes optimized via secondary interfacial

polymerization in ethanol and aqueous systems are hereafter referred to as PA-QPE and PA-QPW, respectively.

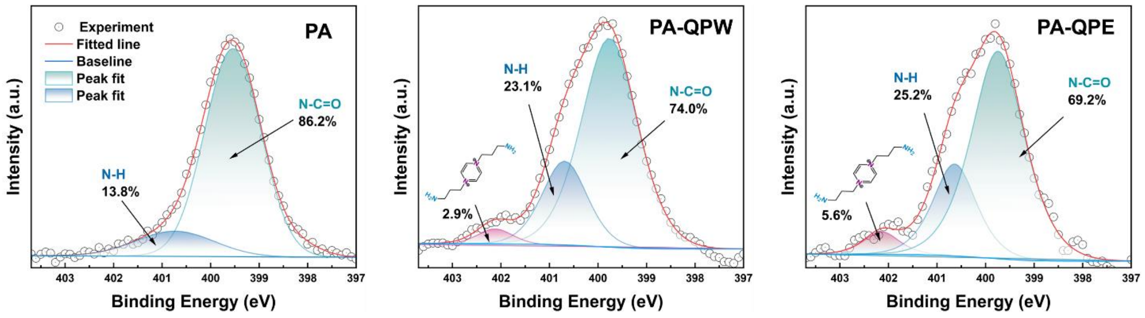

Figure S19 High-resolution N1s XPS spectra of membranes. **Note**: the membranes optimized via secondary interfacial polymerization in ethanol and aqueous systems are hereafter referred to as PA-QPE and PA-QPW, respectively.

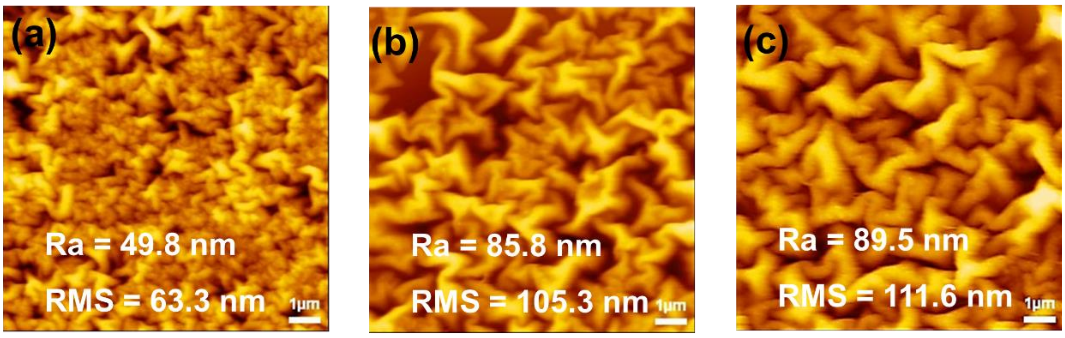

Figure S20. Surface AFM image of (a) the PA, (b) the PA-QPW and (c) PA-QPE membranes. **Note**: the membranes optimized via secondary interfacial polymerization in ethanol and aqueous systems are hereafter referred to as PA-QPE and PA-QPW, respectively.

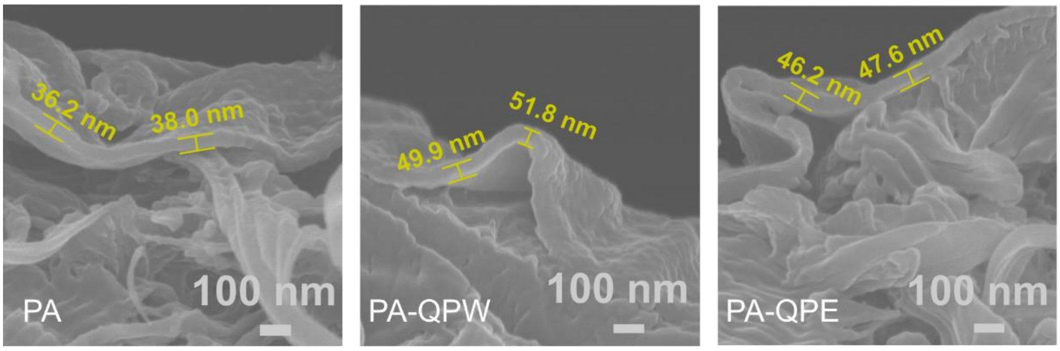

Figure S21. Cross-sectional SEM image of (a) the PA, (b) the PA-QPW, and (c) the PA-QPE membranes. **Note**: the membranes optimized via secondary interfacial polymerization in ethanol and aqueous systems are hereafter referred to as PA-QPE and PA-QPW, respectively.

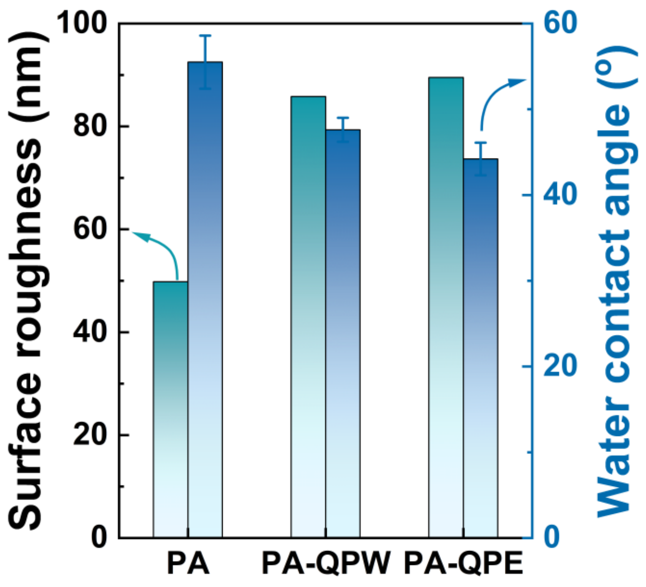

Figure S22 Surface roughness and water contact angle of membranes. **Note**: the membranes optimized via secondary interfacial polymerization in ethanol and aqueous systems are hereafter referred to as PA-QPE and PA-QPW, respectively.

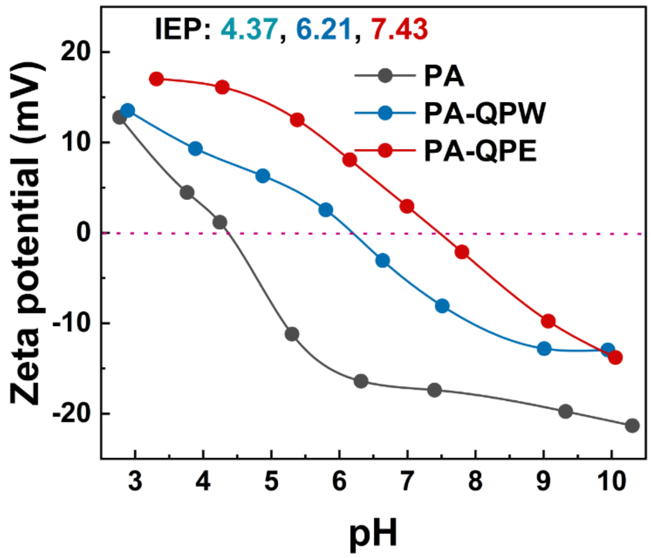

Figure S23 Surface zeta potential of membranes. **Note**: the membranes optimized via secondary interfacial polymerization in ethanol and aqueous systems are hereafter referred to as PA-QPE and PA-QPW, respectively.

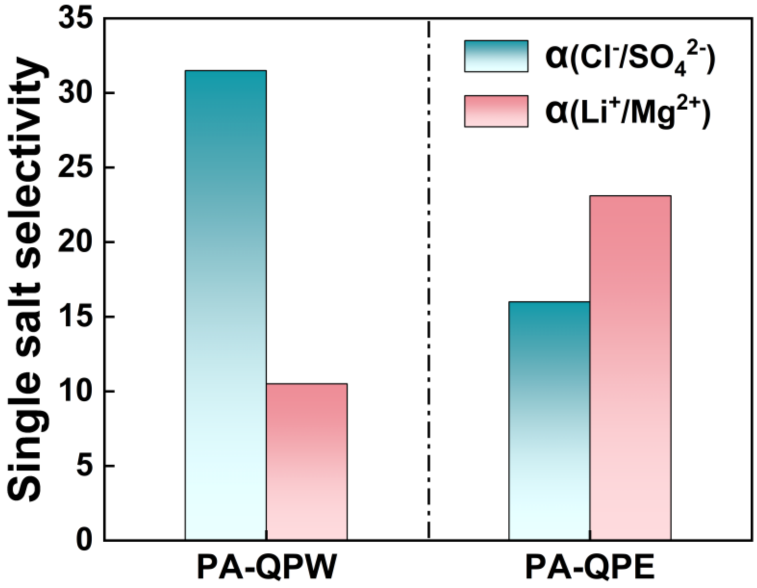

Figure S24 Single salt selectivity of membranes under different solvents

The selectivity reported in Figure S21 represents the ideal selectivity calculated from single-salt filtration data based on the equation rather than results obtained from additional mixed salt experiments. Specifically, the single salt rejections for NaCl, Na_2SO_4, LiCl, and $MgCl_2$ were first measured as described previously, and the corresponding data are summarized in Figures S11 and S16.

For example, the selectivity of Salt A over Salt B is calculated using the following formula:

$$S_{A/B} = \frac{1 - R_A}{1 - R_B}$$

where R_A and R_B represent the single-salt rejection rates for Salt A and Salt B, respectively.

Table S3 Ionic radius, hydrated radius, hydration energy, and separation performance of ions investigated in this study. Note: data was collected from the same literature.

Cations						PA	PA-QPW	PA-QPE
	Ionic Radius (Å)	Stokes Radius (Å)	Hydrated Radius (Å)	Hydration energy (kcal mol^{-1})	Salt used in the test	Average Rejection (%)		
**Co^{2+}**	0.72	3.35	4.23	457.7	$CoCl_2$	97.3	91.7	97
**Mg^{2+}**	0.65	3.47	4.23	437.4	$MgCl_2$	97.9	92.5	96.6
**Cu^{2+}**	0.72	3.25	4.19	480.4	$CuCl_2$	96.5	90.2	95.3
**Ca^{2+}**	0.99	3.1	4.12	359.7	$CaCl_2$	96.9	90.4	94.1
**Ba^{2+}**	1.35	2.9	4.04	298.8	$BaCl_2$	95.3	88.9	94.2
**Li^{+}**	0.6	2.38	3.82	113.5	LiCl	35.9	21.4	23.7
**Na^{+}**	0.95	1.84	3.58	87.2	NaCl	31.2	18.1	21.8
**K^{+}**	1.33	1.25	3.31	70.5	KCl	29.6	16.5	18.9
**Rb^{+}**	1.48	1.18	3.29	65.7	RbCl	26.5	17.3	20.5
**Cs^{+}**	1.69	1.19	3.29	59.8	CsCl	27.1	16.2	21.8

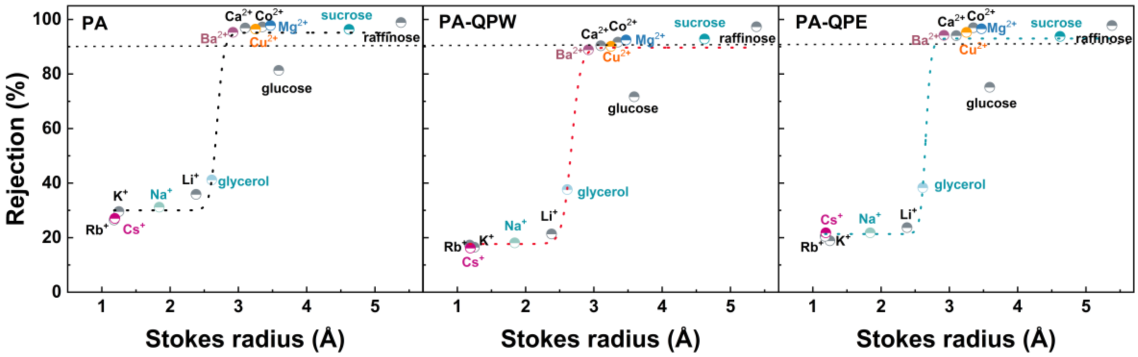

Fig. S25 Retention curves for cations with different stokes radius.

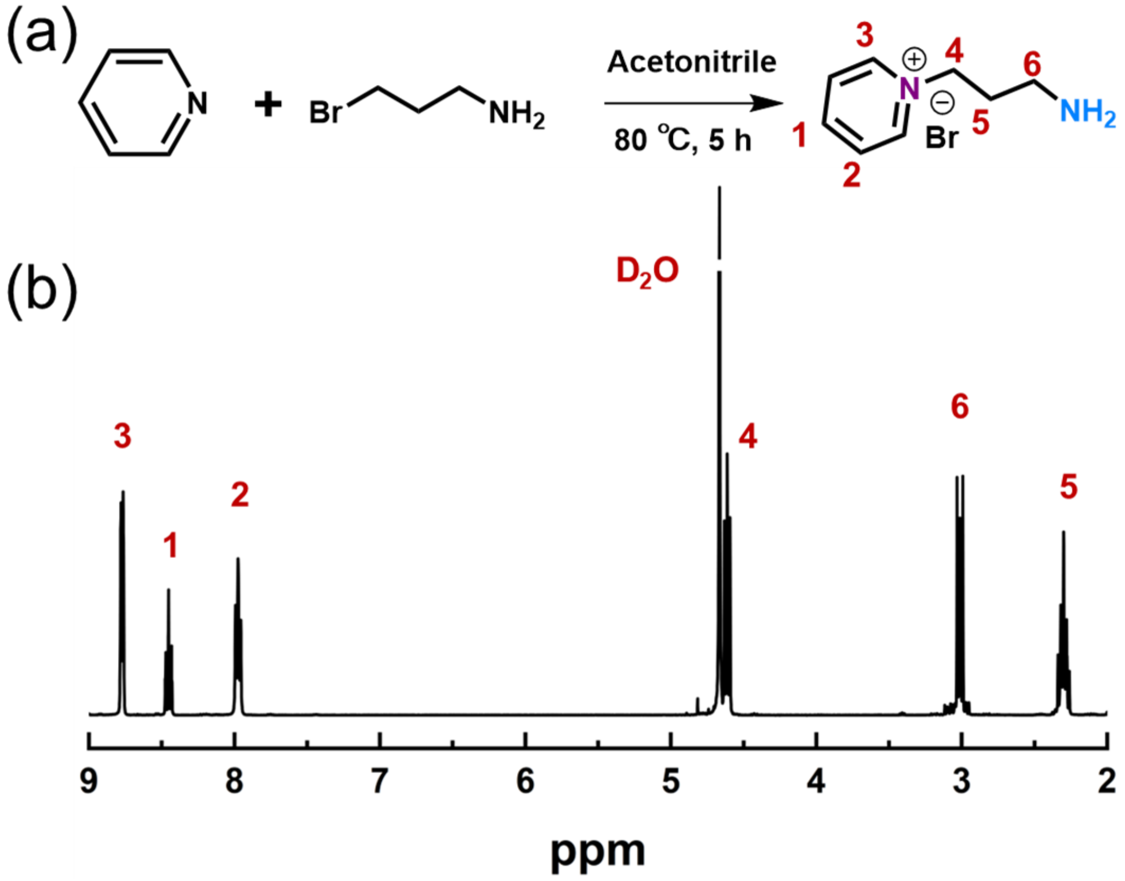

Fig. S26 (a) synthesis reaction and (b) ^{1}H NMR spectra of AP monomer

This compound was synthesized based on the process [6]. Briefly, to a solution of 3-bromopropylammonium bromide (2.0 g, 9.14 mmol) in dry acetonitrile under argon was added dropwise freshly distilled pyridine (4 mL, 49.6 mmol). The reaction mixture was stirred under reflux for 5 h. After cooling to room temperature, the precipitate was collected by filtration, washed with anhydrous ethyl acetate (40 mL), and dried overnight in a desiccator under reduced pressure to obtain 1-(3-ammoniopropyl)pyridinium bromide (AP) as a white solid.

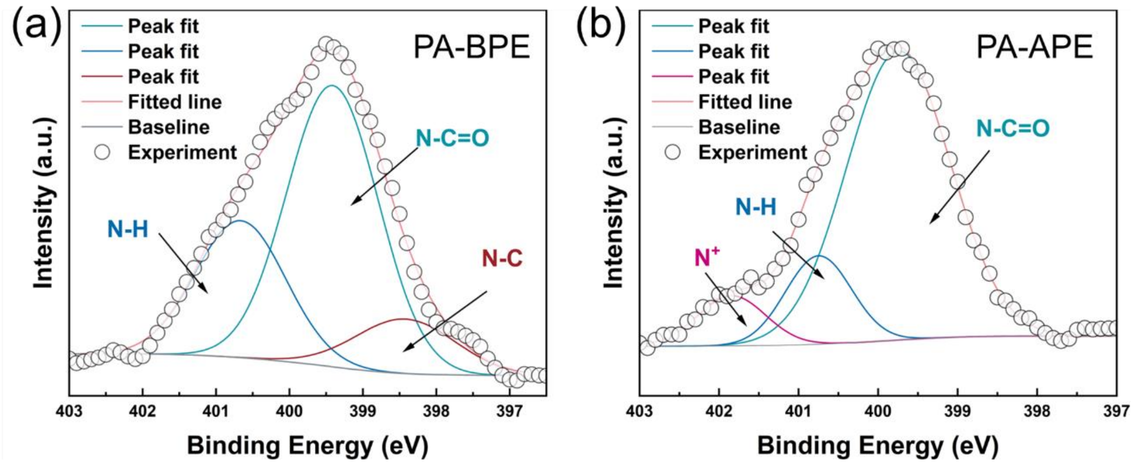

Fig. S27 High-resolution N1s XPS spectra of membranes.

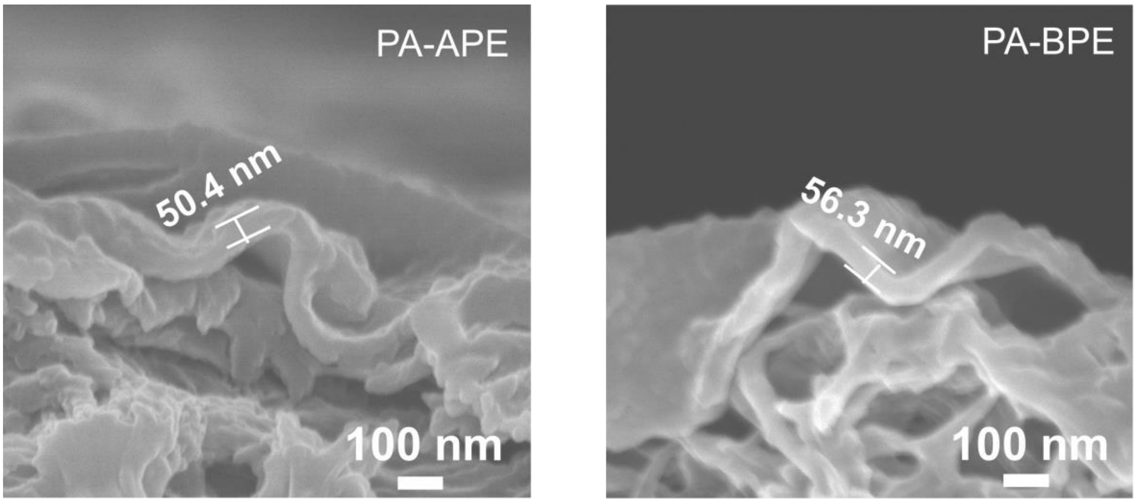

Fig. S28 Cross-sectional SEM image of (a) the PA-APE and (c) PA-BPE membranes.

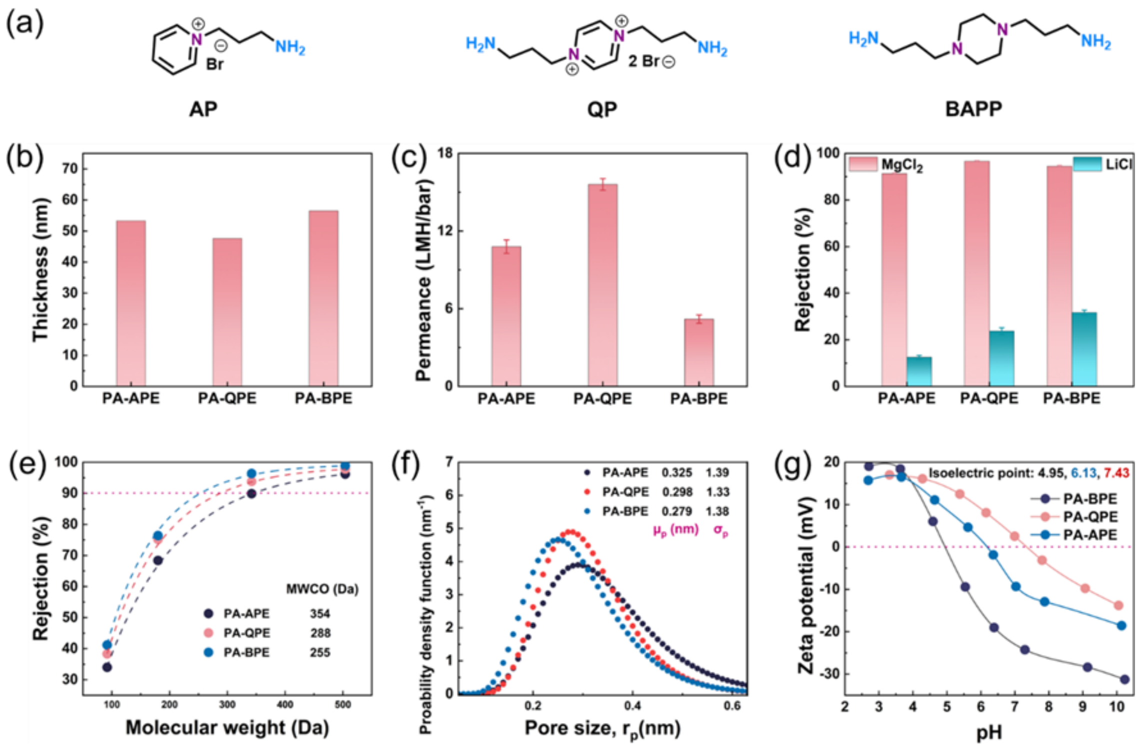

Figure 29. Verification of the bidentical QP strategy. a) Chemical structures of QP and control monomers used for two-stage IP; (b) thickness of active layers; (c) pure water permeance; (d) rejection of $MgCl_2$ and LiCl; (e) MWCO; (f) pore size distribution, and (g) surface zeta potential of different membranes.

The chemical structure of AP was confirmed by 1H NMR (Figure S26), and XPS N1s spectra (Figure S27) verify that both AP and BAPP are covalently incorporated into the PA layer. Although the PA layers formed from these monomers exhibit comparable thicknesses in the 50-60 nm range, their precise thicknesses follow the order PA-BPE (56 nm), PA-APE (50 nm), and PA-QPE (48 nm) (Figure 5b), revealing distinct reaction pathways. BAPP promotes outward second growth with residual acyl chlorides, resulting in thicker PA layers, whereas AP induces only limited regrowth due to its single amine

group. In contrast, QP, influenced by its quaternary ammonium center, primarily undergoes local rearrangement rather than vertical thickening within the nascent PA network, giving rise to the smallest thickness change. Despite the small differences in thickness, water permeability shows an opposite trend of PA-QPE > PA-APE > PA-BPE. The bidentate nature of QP enables effective bridging with residual acyl chlorides, and its preferential localization facilitates the formation of a moderately expanded free volume in the PA layer, yielding the highest permeability. AP provides limited control over network density, whereas the strong condensation reaction of BAPP significantly densifies the network and suppresses water transport. The $MgCl_2$/LiCl separation behavior further reflects these structural differences. All three membranes maintain $MgCl_2$ rejection above 90%, yet LiCl rejection increases from PA-APE to PA-QPE and becomes highest in PA-BPE. The relatively loose PA-APE structure provides insufficient size discrimination for Mg^{2+}, and the overly dense PA-BPE structure imposes strong hindrance toward both ions. PA-QPE maintains high Mg^{2+} rejection while exerting moderate hindrance toward Li^{+}, consistent with a sub-nanometer structure closer to the ideal separation region. MWCO and pore size analyses corroborate these observations PA-APE shows the largest MWCO and pore size, PA-BPE exhibits the smallest values, and PA-QPE lies between these extremes with a moderately dense yet partially expanded structure that aligns with its balanced permeability and selectivity. Surface charge measurements further reveal that QP establishes a more persistent positive charge environment, as indicated by the highest isoelectric point (7.43). AP introduces

quaternary ammonium groups in a more dispersed manner, while BAPP contributes minimally to charge regulation.

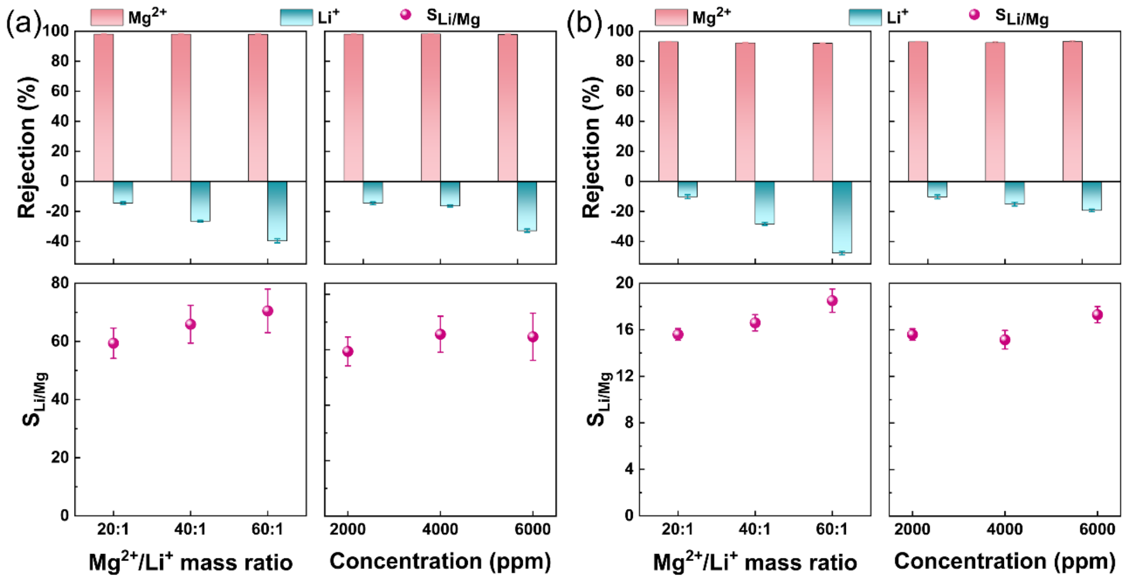

Fig. S30 Ion rejection and selectivity of (a) PA-QPE and (a) PA-QPW as a function of Mg^{2+}/Li^{+} mass ratios and total salt concentration

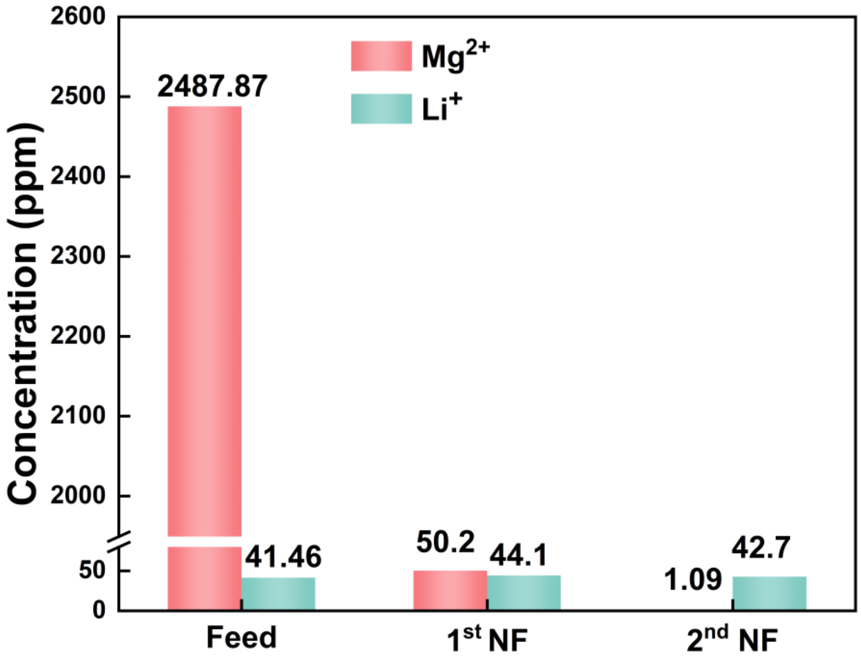

Fig. S31 Two-stage nanofiltration performance for simulated brine with extreme feed conditions (total salt concentration:10000 ppm, Mg^{2+}/Li^{+} mass ratio: 60:1)

Table S4 Li^+/Mg^{2+} separation precision and efficiency of the prepared membrane and other reported NF membranes.

Membranes	P_{Li}	P_{Mg}	Pw	P_{Li}/P_{Mg}	P_{Li}/P_w	Reference
NF90	0.217	0.0042	3.3	51.66667	0.065758	[7]
NF270	3.76	0.847	19.7	4.439197	0.190863	[7]
DL	3.634	0.4845	6.1	7.500	0.5957	[8]
DK	1.47	0.0374	4.51	39.3	0.326	[9]
NFX	0.9753	0.0339	2.1	28.77	0.4644	[10]
PIP-TMC	3.78	0.0307	16	123.127	0.23625	[11]
PIP-TMC	2.95	0.0161	12.7	183.2298	0.232283	[12]
PIP-TMC	2.70	0.0191	8.7	141.3613	0.310345	[13]
PIP-TMC	8.39	0.0715	28.7	117.3427	0.292334	[14]
PIP-TMC	3.23	0.0159	11.5	203.1447	0.28087	[15]
PIP-TMC	2.02	0.03	5.2	67.33333	0.388462	[1]
PIP-TMC	2.65	0.0479	11.1	55.32359	0.238739	[16]
PIP-TMC	2.822	0.105	14	26.87619	0.201571	[17]
PIP-TMC	1.916	0.1075	11.38	17.82326	0.168366	[18]
PEI-TMC	1.0710	0.0109	11.4	98.25688073	0.093947368	[19]
PEI-TMC	0.5685	0.0227	11.2	25.04405286	0.050758929	[20]
PEI-TMC	1.5023	0.0177	9.94	84.87570621	0.151136821	[21]

PEI-TMC	0.898	0.0240	11.24	37.41666667	0.079893238	[22]
PEI-TMC	3.0848	0.1742	23.1	17.70838117	0.133541126	[23]
PEI-TMC	0.5435	0.0061	9.19	89.09836066	0.05914037	[24]
PEI-TMC	2.5196	0.0965	34.3	26.10984456	0.073457726	[25]
Cu-MPD	1.219	0.1428	10.36	8.536414566	0.117664093	[26]
Ag@ZFZ-GO	0.0934	0.001	44.37	93.4	0.002105026	[27]
Polyester	1.5176	0.0605	16.7	25.08429752	0.090874251	[28]
PA-QPE	5.21	0.0273	15.6	190.8425	0.333974	This work
PA-QPW	4.00	0.1014	12.3	39.44773	0.325203	This work

References

[1] P. Xu, S. Duan, Z. Li, M. Hu, P. Zhang, L. Dai, Z. Mai, K. Guan, H. Matsuyama, *Advanced Functional Materials* **2024**, *n/a*, 2416458.

[2] P. Xu, R. R. Gonzales, J. Hong, K. Guan, Y.-H. Chiao, Z. Mai, Z. Li, S. Rajabzadeh, H. Matsuyama, *J. Membr. Sci.* **2023**, *668*, 121251.

[3] Q. Song, Y. Lin, T. Ueda, Q. Shen, K.-R. Lee, T. Yoshioka, H. Matsuyama, *J. Membr. Sci.* **2022**, *657*, 120679.

[4] Y. Lin, X. Yao, Q. Shen, T. Ueda, Y. Kawabata, J. Segawa, K. Guan, T. Istirokhatun, Q. Song, T. Yoshioka, H. Matsuyama, *Nano Lett.* **2021**, *21*, 6525.

[5] Q. Shen, Y. Lin, T. Ueda, P. Zhang, Y. Jia, T. Istirokhatun, Q. Song, K. Guan, T. Yoshioka, H. Matsuyama, *Journal of Membrane Science* **2022**, *646*, 120269.

[6] Y. Li, Z. Jiang, Z. Liu, B. Li, *Langmuir* **2024**.

[7] B. K. Pramanik, M. B. Asif, S. Kentish, L. D. Nghiem, F. I. Hai, *J. Environ. Chem. Eng.* **2019**, *7*, 103395.

[8] S.-Y. Sun, L.-J. Cai, X.-Y. Nie, X. Song, J.-G. Yu, *J. Water Process Eng.* **2015**, *7*, 210.

[9] Y. Li, Y. Zhao, H. Wang, M. Wang, *Desalination* **2019**, *468*, 114081.

[10] R. Wang, R. He, T. He, M. Elimelech, S. Lin, *Nat Water* **2023**, *1*, 291.

[11] K. Chen, S. Zhao, H. Lan, T. Xie, H. Wang, Y. Chen, P. Li, H. Sun, Q. J. Niu, C. Yang, *J. Membr. Sci.* **2022**, *660*, 120860.

[12] P. Hu, B. Yuan, Q. J. Niu, K. Chen, Z. Xu, B. Tian, X. Zhang, *Desalination* **2022**, *527*, 115553.

[13] S. Zhao, W. Cui, Q. Shen, Z. Yao, C. Fang, L. Zhang, L. Zhu, *J. Membr. Sci.* **2024**, *690*, 122207.

[14] B. Yuan, M. Wang, M. Wu, D. Yang, K. Zhang, S. Zhao, Y. Zhang, P. Hu, M. You, S. Zhao, K. Chen, X. Zhang, J. Jiang, X. Lou, Q. J. Niu, *J. Membr. Sci.* **2024**, *701*, 122743.

[15] S. Zhao, L. Dai, Z. Mai, B. Li, P. Zhang, M. Zhang, A. Matsuoka, K. Guan, R. Takagi, H. Matsuyama, *Advanced Science n/a*, 2500255.

[16] Z. Yang, W. Fang, Z. Wang, R. Zhang, Y. Zhu, J. Jin, *J. Membr. Sci.* **2021**, *620*, 118862.

[17] H.-Z. Zhang, Z.-L. Xu, H. Ding, Y.-J. Tang, *Desalination* **2017**, *420*, 158.

[18] H. Li, Y. Li, M. Li, Y. Jin, G. Kang, Y. Cao, *J. Membr. Sci.* **2023**, *669*, 121321.

[19] X. Li, M. Xu, X. Liu, Q. She, W. J. Lau, L. Yang, *Water Research* **2025**, 123400.

[20] P. Xu, J. Hong, X. Qian, Z. Xu, H. Xia, Q.-Q. Ni, *Desalination* **2020**, *488*, 114522.

[21] W. Ji, Y. Li, F. Duan, L. Liu, R. Cao, J. Yin, H. Sun, J. Luo, H. Cao, *Journal of Membrane Science* **2025**, *729*, 124160.

[22] Y. Yan, J. Wang, W. Wang, C. Han, *Journal of Membrane Science* **2025**, *721*, 123812.

[23] Y. Fan, H. Tian, K. Wang, G. Zhou, J. Wang, G. Li, Y. Cao, Y. Wang, X. Jiang, W. Kou, *Separation and Purification Technology* **2025**, *358*, 130276.

[24] J. Wei, X. Ma, F. Yang, S. Jia, Z. Wang, *Journal of Membrane Science* **2025**, *728*, 124135.

[25] H. Peng, X. Liu, Y. Su, J. Li, Q. Zhao, *Angew. Chem. Int. Ed.* **2023**, *62*, e202312795.

[26] L. Wang, D. Rehman, P.-F. Sun, A. Deshmukh, L. Zhang, Q. Han, Z. Yang, Z. Wang, H.-D.

Park, J. H. Lienhard, C. Y. Tang, *ACS Appl. Mater. Interfaces* **2021**, *13*, 16906.
[27] Y. Hao, X. Liu, Y. Zhang, X. Zhang, Z. Li, X. Chen, *Advanced Science* **2024**, *11*, 2406535.
[28] J. Li, H. Peng, K. Liu, Q. Zhao, *Advanced Materials* **2024**, *36*, 2309406.
